# Improved aboveground biomass estimation and regional assessment with aerial lidar in California’s subalpine forests

**DOI:** 10.1186/s13021-024-00286-w

**Published:** 2024-12-20

**Authors:** Sara Winsemius, Chad Babcock, Van R. Kane, Kat J. Bormann, Hugh D. Safford, Yufang Jin

**Affiliations:** 1 Department of Land, Air and Water Resources, University of California, Davis, CA 95616 USA; 2 Department of Forest Resources, University of Minnesota, St. Paul, MN 55108 USA; 3 School of Environmental and Forest Sciences, University of Washington, Seattle, WA 98195 USA; 4 Airborne Snow Observatories, Inc., Mammoth Lakes, CA 93546 USA; 5 Department of Environmental Science and Policy, University of California, Davis, CA 95616 USA; 6 Vibrant Planet, Incline Village, NV 86451 USA

**Keywords:** Remote sensing, Vegetation structure, Carbon monitoring, Bayesian hierarchical spatial modeling, Aboveground biomass, Subalpine forests

## Abstract

**Background:**

Understanding the impacts of climate change on forest aboveground biomass is a high priority for land managers. High elevation subalpine forests provide many important ecosystem services, including carbon sequestration, and are vulnerable to climate change, which has altered forest structure and disturbance regimes. Although large, regional studies have advanced aboveground biomass mapping with satellite data, typically using a general approach broadly calibrated or trained with available field data, it is unclear how well these models work in less prevalent and highly heterogeneous forest types such as the subalpine. Monitoring biomass using methods that model uncertainty at multiple scales is critical to ensure that local relationships between biomass and input variables are retained. Forest structure metrics from lidar are particularly valuable alongside field data for mapping aboveground biomass, due to their high correlation with biomass.

**Results:**

We estimated aboveground woody biomass of live and dead trees and uncertainty at 30 m resolution in subalpine forests of the Sierra Nevada, California, from aerial lidar data in combination with a collection of field inventory data, using a Bayesian geostatistical model. The ten-fold cross-validation resulted in excellent model calibration of our subalpine-specific model (94.7% of measured plot biomass within the predicted 95% credible interval). When evaluated against two commonly referenced regional estimates based on Landsat optical imagery, root mean square error, relative standard error, and bias of our estimations were substantially lower, demonstrating the benefits of local modeling for subalpine forests. We mapped AGB over four management units in the Sierra Nevada and found variable biomass density ranging from 92.4 to 199.2 Mg/ha across these management units, highlighting the importance of high quality, local field and remote sensing data.

**Conclusions:**

By applying a relatively new Bayesian geostatistical modeling method to a novel forest type, our study produced the most accurate and precise aboveground biomass estimates to date for Sierra Nevada subalpine forests at 30 m pixel and management unit scales. Our estimates of total aboveground biomass within the management units had low uncertainty and can be used effectively in carbon accounting and carbon trading markets.

**Supplementary Information:**

The online version contains supplementary material available at 10.1186/s13021-024-00286-w.

## Introduction

Subalpine forests, which are found in mountainous regions worldwide, grow at high elevations up to alpine treeline ecotones where it becomes too cold for tree growth. These forests provide many essential ecosystem services including water provision, climate refugia, animal and plant habitat, and carbon storage [66, 78, 98]. They are also considered early-warning indicators of ecosystem biome shifts due to their sensitivity to climate change [44]. Changes to AGB in subalpine forest ecosystems from increased temperatures include infilling, some upslope advance, and greater disturbance prevalence [2, 21, 100], making it unclear whether these forests will provide more or less carbon sequestration in the future.

Subalpine forests worldwide face mounting challenges from climate change and related increases in disturbance frequency and severity [2, 45, 53]. Abiotic and biotic disturbances due to cold, harsh climates, avalanches, erosion, insects, and more are omnipresent in subalpine forests [45]. In some regions such as the Alps, recent changes in pastoral use and afforestation efforts have increased forest cover [6], and in the Tibetan Plateau, models predict forest expansion due to increased temperatures [64]. The intensifying impacts of climate change in subalpine forests make understanding AGB pressing.

Structural changes in subalpine forests of the Sierra Nevada, California exemplify trends seen worldwide. Increasing temperature, especially at night, has decreased spring snowpack by up to 70% in the northern and central Sierra Nevada, increasing drought stress [19, 39, 95]. The associated multi-week extensions of the growing season have altered forest structure despite minimal human management (e.g., fire suppression and logging) [76, 95]. These changes include increases in small tree density [21], increasing spread of bark beetles and white pine blister rust [25, 74, 114], increasing proportion of high severity fire [70], and the increasing upper elevation of fire [2, 96]. Upward encroachment of montane species such as red fir and Jeffrey pine into subalpine forests plus greater survival rates for seedlings could result in greater density along with heightened extinction risks for subalpine tree species [9, 15, 41, 78, 94]. These changes in climate and disturbance regimes, detailed for the Sierra Nevada but which are seen globally [6, 45], have made AGB estimation of great interest to land managers, researchers, and agencies [47, 50].

**Fig. 1:**
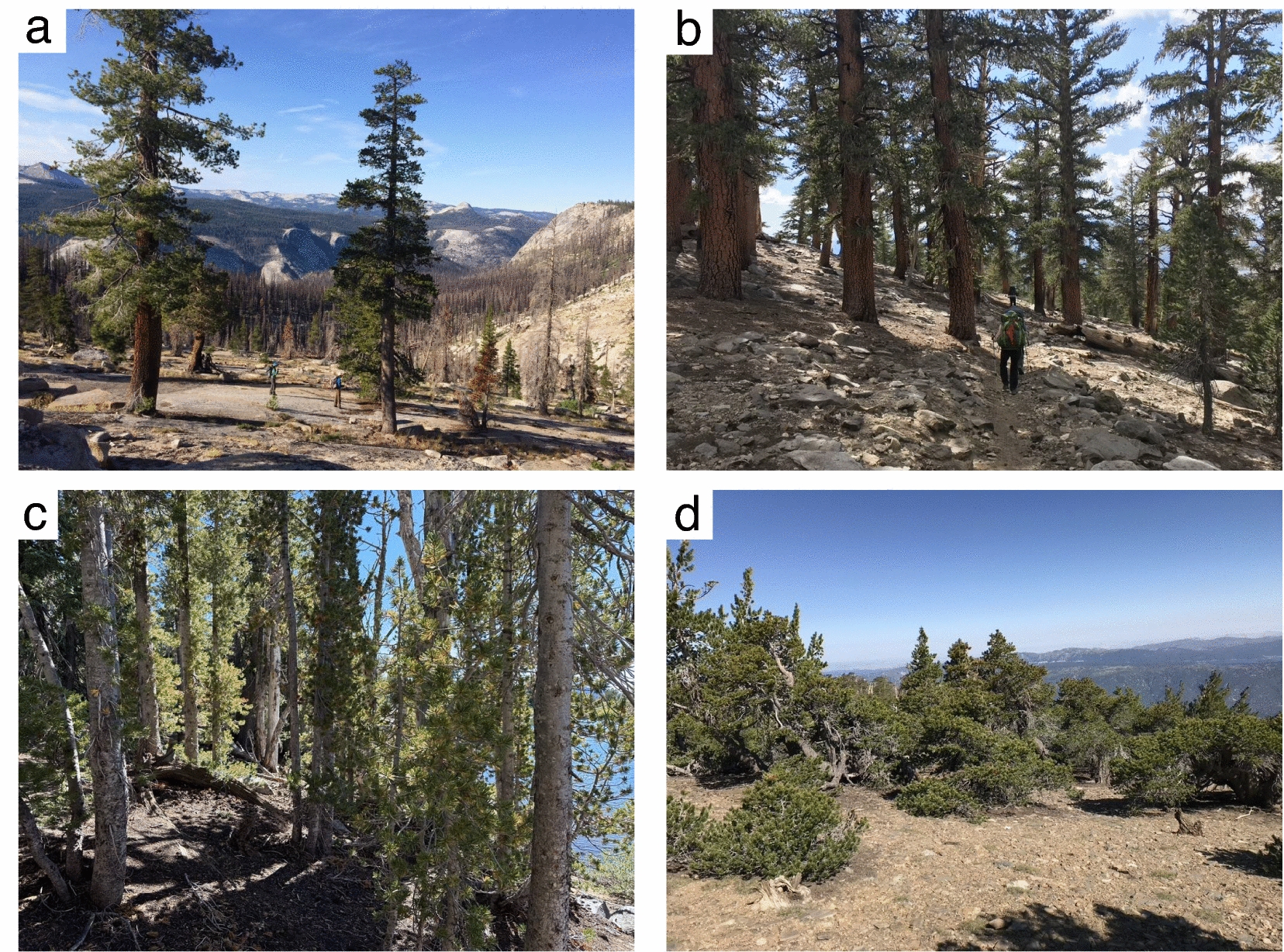
Examples of some of the forest structures commonly found in the Sierra Nevada subalpine region: **a** Open subalpine with large trees in granite, **b** large trees in more continuous forest, **c** tight canopy of small, thin trees, **d** windswept, short trees. Photos by S. Winsemius

Subalpine landscapes often consist of a mosaic of relatively small forest and woodland patches along with rock outcrops, meadows, shrubs, and riparian ecosystems in an often steep region with high fine-scale topographic complexity [34, 44, 76, 88]. These regions are limited by low temperature and geomorphic characteristics, and forest distribution and structure are controlled by gradients in temperature, wind, seasonal precipitation, and snowpack duration [44, 61, 69, 106]. Forest patches vary in tree size, density, and canopy cover (Fig. 1). Heterogeneous regions may need relatively higher plot densities to represent variation in forest types [112]. Yet, subalpine forests are typically undersampled due to difficult access and the high proportion of sparse woodland and non-forest areas.

Combining remote sensing data and high-quality field reference data allows improved modeling of AGB in regions characterized by diverse forest structures [38, 116]. Optical satellite imagery is readily available with long time series, full spatial coverage, high spatial resolution (< 30 m), and frequent revisits (< 16 days) [89], enabling streamlined monitoring of change over time [58]. However, spectral reflectance is limited in its capacity to predict AGB due to saturation at high canopy density and the inability to observe 3-dimensional forest structure [116]. Many methods have been used with these data to estimate AGB. Recently, machine learning methods such as random forests have become common due to flexible use and reductions in bias and overfitting [14, 49, 89]. Many machine learning approaches do not provide the capability to assess uncertainty for regions, which is important for decision making in forest management.

Because of their more limited distribution, aboveground biomass (AGB) in subalpine forests has often been estimated from satellite data using models developed for areas dominated by lower- or mid-elevation forests in larger regional projects [8, 49, 58]. However, accuracy from broad, regional models is unknown in the subalpine. Subalpine forests represent a relatively small area in national and regional models and have correspondingly fewer plots for model calibration and training. They also have different forest structures and complex terrain that may lead to different relationships between biomass and predictor variables than dominant forest types.

Active sensor data such as lidar and synthetic aperture radar (SAR) have quickly become key data sources for AGB models because they capture forest structure at plot, stand, and landscape levels [55, 62] and thus reduce AGB estimation error significantly compared with passive optical data [116]. Spaceborne missions such as NASA’s Global Ecosystem Dynamics Investigation (GEDI) [23], NASA’s Ice Cloud and land Elevation Satellite (ICESat-2) [1], and the NASA-Indian Space Research Organization (ISRO) Synthetic Aperture Radar (NISAR) [57] are all applied to AGB mapping at large scales. While these present cutting-edge technology with many uses, the lidar projects (ICESat-2 and GEDI) collect data in sampling schemes with wide footprints, and they typically have moderate spatial resolution [27], which cannot capture fine-scale heterogeneity as seen in subalpine forests. Application in subalpine regions is also challenged by higher errors on steep slopes (ICESat-2 and NISAR) and underestimation at higher AGB values [27].

High resolution airborne lidar can provide high quality data for AGB estimates [24, 111], but regions with available data are limited [30], and revisits are typically infrequent [29], including in high elevation regions. AGB estimation in the subalpine has thus been spatially limited [22, 63, 97] or subsumed in larger regional studies [8, 58]. These regional studies, however, use training data dominated by lower elevation plots and often use optical data rather than lidar [48, 58]. A comparison of broad-scale AGB maps from spaceborne and airborne lidar and SAR in mangroves, another specialized forest type, found local calibration was important for calculating area-wide AGB, especially in shorter forests [102]. We posit that an explicit focus on the subalpine, with additional data and a model tuned for this region, will allow us to improve AGB estimates compared with other state- or larger regional models currently available.

Many current AGB modeling efforts lack rigorous uncertainty measures at scales relevant for both local management decisions and policy. Spatial modeling improves AGB prediction by incorporating spatial position and autocorrelation to avoid inflated accuracy estimates [18] and by reporting rigorous uncertainty measurements. Geostatistical methods enable valid model-based statistical inference, making them valuable in a monitoring, reporting and verification context. Geostatistical methods provide an excellent model framework because they account for extraneous spatial autocorrelation, which helps us better accommodate the statistical assumptions of model-based inference [3, 28]. In this model framework, estimates and errors can be obtained at the pixel level and within areas such as management units (MUs). Pixel level maps can help guide local management decisions, while MU estimates have lower errors and may be more useful for AGB accounting and policy decisions [10, 58, 68]. In this study we employ this model framework with field-measured AGB as the response variable, lidar metric predictors, and a spatial error term. It is computationally difficult to estimate parameters in a geostatistical model using standard frequentist methods, but there has been a lot of progress toward estimating model parameters more rigorously using Bayesian approaches [4]. This ensures that autocorrelation is taken into account so that standard deviations and credible intervals are not inflated at the pixel level or at the MU level. We obtain statistically rigorous uncertainty estimates at regional and pixel levels, which many AGB estimation studies lack [13, 116].

In this study, we aim to demonstrate the potential for improved estimation and uncertainty assessment of live and dead tree AGB in subalpine forests and conduct a thorough subalpine AGB assessment across scales. We combine multiple unique field datasets and aerial lidar surveys in Sierra Nevada subalpine forests. Specifically, we ask the following questions: (1) How accurately and precisely can we estimate subalpine forest AGB using aerial lidar surveys and field data in a Bayesian geostatistical model, relative to field data and to two previous regional AGB mapping efforts? (2) What is the distribution of subalpine forest AGB in the Sierra Nevada? and (3) How does AGB vary across management units?

## Methods

### Study area

Subalpine forests are found along the Sierra Nevada crest, above upper montane forests (dominated by *Abies magnifica* [red fir]) and below the treeline isotherm, where temperatures are too cold to support tree growth [34]. They are distributed across elevations of approximately 2400–3100 m in the northern Sierra Nevada and 2750–3500 m in the south [77]. Subalpine forests are found in a patchy mosaic with actual forest cover typically comprising less than 50% of the landscape surface [34]. Dominant species include southern foxtail pine (*Pinus balfouriana*), whitebark pine (*P. albicaulis*), mountain hemlock (*Tsuga mertensiana*), limber pine (*P. flexilis*), lodgepole pine (*P. contorta* ssp. *murrayana*), western white pine (*P. monticola*), and Sierra juniper (*Juniperus grandis*).

Each of the species and forest types have distinct characteristics, described in detail in Fites-Kaufmann et al. [34]. Many species have a shrubby krummholz growth form at their upper elevations, including whitebark pine, limber pine, and mountain hemlock; treeline areas of the Sierra Nevada are dominated by whitebark pine in the north, limber pine co-occurring with lodgepole pine in the central region, and foxtail pine to the south, with foxtail pine remaining as a short, squat upright tree in low density stands. All these species can grow as large trees under favorable conditions, epitomized by western white pine at up to 40 m in height [88]. At lower elevations, western white pine, lodgepole pine, and foxtail pine mix with Jeffrey pine and red fir in denser stands, while harsher environments more often see one or two species co-occurring with lower density due to cold limitation [39]. Sierra juniper in particular is often found growing out of rock fissures.

**Fig. 2:**
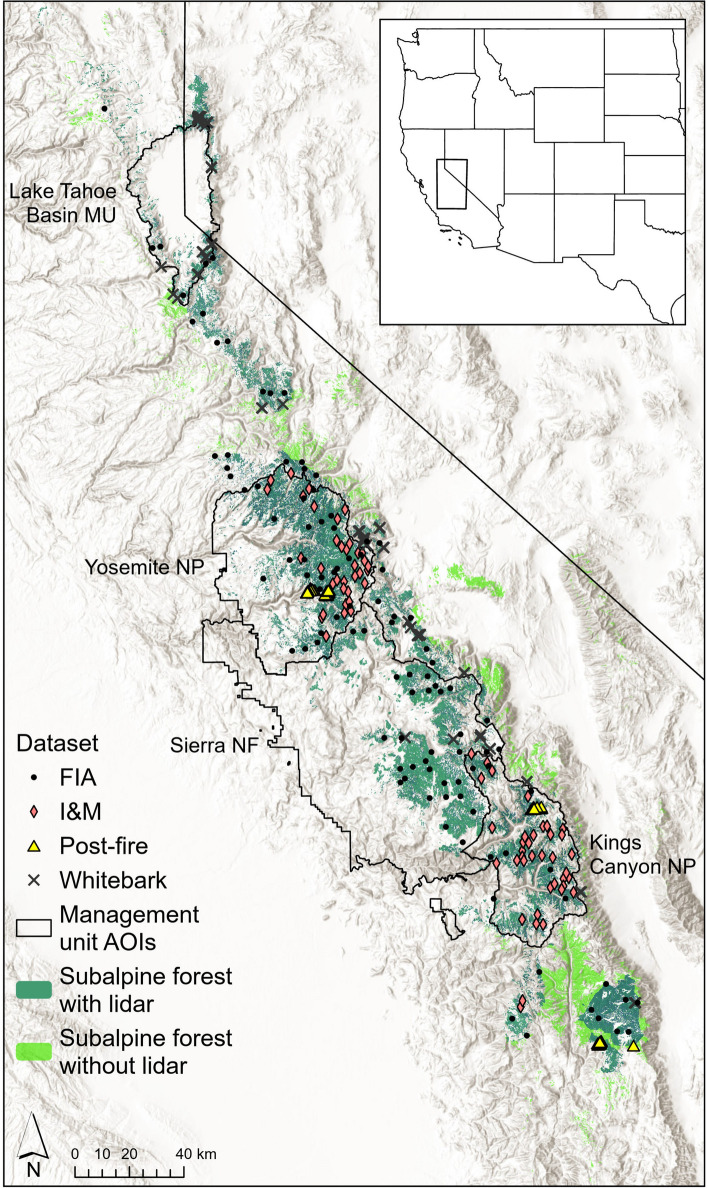
Study area with subalpine forest area from EVeg Existing Vegetation. Areas in dark green have lidar coverage and are included in the model, while light green areas are excluded due to absence of lidar for this study. Points indicate plot locations from four different sampling efforts, and black polygons show the four management unit (MU) areas of interest (AOIs) used in regional predictions

We focused on the subalpine forest types as delineated by the Existing Vegetation (EVeg) maps generated by the USDA Forest Service Pacific Southwest Region [109] using the Classification and Assessment with Landsat of Visible Ecological Groupings (CALVEG) classification system [110]. For this study, subalpine forest types include those indicated in the EVeg maps as the following dominant CALVEG “alliances” in the North Sierran, South Sierran, and Great Basin (Sierra Nevada region only) mapping zones: foxtail pine, lodgepole pine, mountain hemlock, limber pine, subalpine conifers, whitebark pine, western (mountain) juniper, and western white pine (Fig. 2).

### Data

Subalpine forests in the Sierra Nevada are structurally heterogeneous [34, 94] meaning that the number of plots needed to capture the spread of forest structure is relatively high [112]. AGB in subalpine forests appears to be relatively understudied, with spare plot datasets and poor airborne lidar coverage compared to lower elevation forests. We therefore compiled multiple field and lidar datasets to obtain the maximum coverage of subalpine forest distribution and forest types throughout the Sierra Nevada (Fig. 2). These datasets are described in more detail below.

#### Field data

##### Forest inventory and analysis (FIA)

Created by the United States Forest Service (USFS) to monitor forests in a consistent way across the U.S. ([99], p. 200), FIA data form the basis for many regional, state, and country-wide AGB mapping efforts in the nation (e.g., [48, 58, 89]). The FIA program collects data from plot locations that are randomly selected within a grid system, with one plot per 2428 ha [7]. Except under special circumstances, plot locations in non-forest areas or in areas with unsafe access are not sampled, which means that regions with extreme topographic relief are relatively undersampled.

In this study we used 104 FIA plots, which were selected based on the presence of subalpine tree species (southern foxtail pine, whitebark pine, lodgepole pine, limber pine, western white pine, mountain hemlock, and Sierra juniper), location in the Sierra Nevada, elevation above 2200 m, high elevation forest type (delineated by field crews; we excluded the mixed conifer, white fir, and red fir forest types), and collocation of lidar data. Plots were sampled between 2011–2016 (Table 1). For all live and dead trees, crews record species, diameter at breast height (DBH, measured at 1.37 m height), and height, and an associated trees per acre adjustment based on nested plot sizes allows trees to be aggregated to the plot level [108].

**Table 1:** Plot data sources and the number of plots sampled in each year

Name (agency)	Years (number of plots)	Plot size (hectare)	AGB mean (SD) (Mg/ha)
Forest Inventory and Analysis (U.S. Forest Service)	2011 (18), 2012 (20), 2013 (16), 2014 (19), 2015 (13), 2016 (16)	0.0054 ha, 0.067 ha, or 0.1 ha depending on tree DBH (< 12.4 cm, ≥ 12.4 cm, and ≥ 61 cm, respectively)	125.0 (107.0)
Region 5 Whitebark Pine Inventory and Monitoring (U.S. Forest Service)	2013 (2), 2014 (13), 2017 (37), 2018 (21), 2019 (22)	0.08 ha	57.3 (54.2)
Sierra Nevada Inventory & Monitoring Network, White Pine Forest Dynamics Monitoring (National Park Service)	2015 (1), 2016 (22), 2017 (20), 2019 (26), 2021 (1)	0.25 ha	77.3 (78.3)
Post-Fire Subalpine Monitoring (University of California, Davis)	2018 (42), 2019 (51)	0.063 ha	138.0 (54.2)

##### Region 5 whitebark pine inventory and monitoring

Whitebark pine-dominated plots were additionally represented by data from the USFS Region 5 whitebark pine inventory and monitoring program, which began in 2011 with the goal of monitoring whitebark pine mortality [75, 98]. We used 95 plots from this dataset which were co-located with lidar information (Table 1). In this monitoring protocol, plots were stratified in elevation zones across an 800 m elevational belt and locations were chosen for accessibility, relatively uniform slope and access within the plot, and the presence of whitebark pine. Within each general location, multiple plots were randomly selected that were at least 15 m from trails and that were at least 100 m apart [75, 98]. Within the 16.1 m radius plots (0.08 ha), all live and dead trees greater than or equal to 7.6 cm DBH were measured and species, DBH, and height of each tree was recorded. Because whitebark often grows in clusters of similar sized stems, with individuals difficult to discern, crews sometimes recorded clusters of trees with an average DBH and height and a count of how many stems were within the cluster. In our data processing, these counts were expanded, and each member of the cluster was assigned the same DBH and height.

##### Sierra Nevada Inventory & monitoring network

The National Park Service (NPS) Inventory and Monitoring (I&M) White Pine Forest Dynamics Monitoring program is a dataset focused on monitoring of whitebark and foxtail pine stands, from which we used 70 plots with collocated lidar data. The NPS collects data across multiple parks with a common set of monitoring objectives, procedures, and protocols [73, 81]. We used data from Yosemite National Park and Sequoia and Kings Canyon National Parks, collected from 2015 through 2021 (Table 1). Plots are established according to a Generalized Random Tessellated Stratified algorithm [101], and they are revisited on a 3-year resampling frame. For remeasured plots, we used the measurement year that aligned best with the lidar collection year. The quarter hectare (50 × 50 m) macroplots contain measurements for every living tree > 1.37 m tall and every standing dead tree > 5 cm DBH, with data for species, DBH, and height.

##### Post-fire subalpine monitoring

Fires and other disturbances are increasingly affecting high elevation forests, but the representation of disturbed sites in FIA and the two white pine datasets is minimal. To include burned forests in this analysis we conducted field work in 2018 and 2019 and sampled plots across 6 fires that burned in subalpine forests between 2002–2014; 93 plots sampled in this effort were collocated with lidar data. We identified fires that had a full range of fire severity classes (unburned, 0–25, 25–50, 50–75, 75–90, and 90–100% basal area mortality) using the relativized differenced normalized burn ratio [79]. Fires were also selected for accessibility within a 2-day hike from a trailhead. Time-since-fire ranged from 4 to 17 years. We used a high precision GPS (Trimble GeoExplorer 6000 in 2018 and Trimble R1 in 2019) to record the location of each plot. A stratified random sampling design placed 14.2 m radius circular plots (0.063 ha) at the crosshairs of a 200 × 200 m grid, with stratification across fire severity classes and aspect [15]. We measured all standing live and dead trees with DBH > 7.6 cm, and recorded DBH, height, and species.

##### Aboveground biomass (AGB) estimation from plot data

AGB was calculated using the Component Ratio Method (CRM), following the allometric method used by FIA [113] and used in other regional datasets (see below, “Comparisons with other AGB map products” section). This method produces estimates for dry AGB of wood and bark from the ground to the tip, including branches but not foliage (noted as DRYBIO_AG in the FIA database), as is common in most other studies [116]. We modeled AGB from both live and dead trees because of the very slow rate of decay in subalpine forests and the important role of persistent dead AGB [60, 68, 90]. For consistency with FIA data, AGB for non-FIA plots was calculated by a member of the FIA team using the FIA standard protocol (Dr. Andrew Gray, personal communication, November 8, 2022. About 4% of trees in non-FIA datasets lacked measured heights, for these we estimated AGB using allometry from only DBH based on the most relevant equation by species specificity, region of study, and DBH range [16, 52, 68, 87]. AGB of trees over 7.6 cm DBH (the largest common minimum across plots) was summed at the plot level and divided by the plot size to get plot AGB in Mg/ha.

#### Lidar data and preprocessing

Lidar data were not available for the full study area. However, by combining three publicly available datasets (Kern Plateau, Merced watershed, and Tuolumne watershed) along with data from Airborne Snow Observatories, Inc. (ASO), we obtained data over the majority of the study region (Table S1). Lidar data were collected in snow-off months between 2011 and 2021, with most collected between 2018–2021. A Riegl VQ1560 (or VQ1560i or VQ1560ii-s) was used for all but one acquisition, and pulse densities range from 2–27 pls/m^2^. These data were used to create raster layers of area metrics and extract plot-level metrics, which were used to model the relationship between AGB and structure variables and to then estimate AGB at 30 m resolution.

At the time of data processing through June 2022, high elevation forests had limited coverage of publicly available lidar acquisitions in the Sierra Nevada. ASO collects summer snow-off lidar data in some years as part of annual snowpack monitoring [85]. These data cover most of the watersheds in the Sierra Nevada, with high coverage of subalpine forests. ASO and Watershed Sciences provided digital terrain models at 3 and 1 m resolution, respectively.

We processed the lidar data into raster grid formats using the raster and terra packages in R [42, 43, 91] and the AreaProcessor workflow tool within the FUSION software package [72]. We calculated all metrics at 30 m resolution to match with other AGB maps commonly produced for the larger bioregion and to minimize gaps in the outputs due to low point density in some areas. Rasters were clipped to the CALVEG subalpine forest types listed above (“Study area” section). We masked 3% of pixels that had very tall heights from the inputs because subalpine trees in the Sierra Nevada do not grow that tall: we excluded pixels with 95th percentile height greater than 64 m or mean height greater than 40 m (the tallest tree measured in any plot was 54 m, and 99% of 95th percentile heights were below 32 m; > 99.9% of mean heights were below 30 m). These tall heights are often artifacts caused by small horizontal inaccuracies which are exacerbated by steep topographic features such as cliffs.

**Fig. 3:**
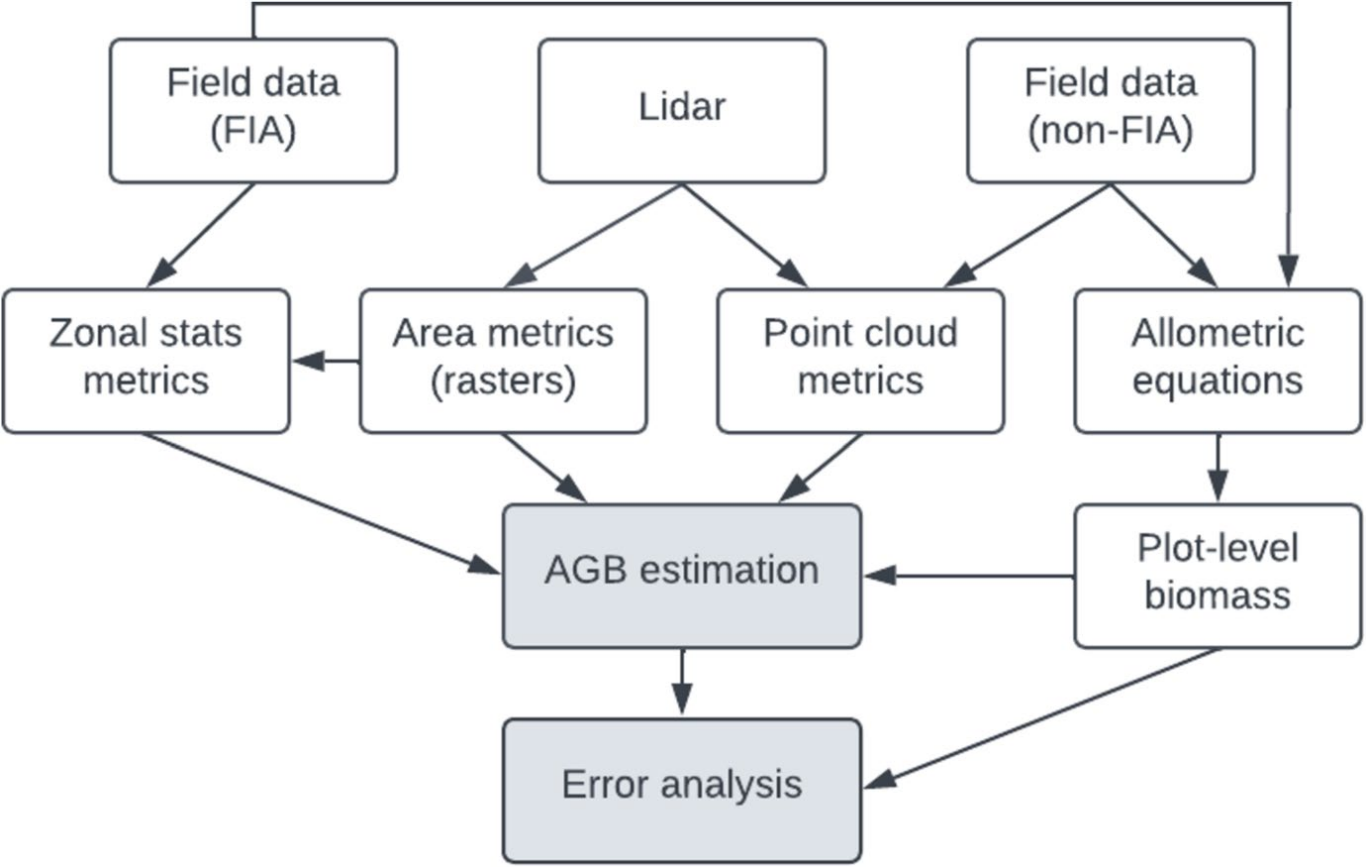
Flowchart showing the flow of input data through processing to the model and error analysis

To build the relationship between plot-based AGB with aerial lidar metrics, we extracted the lidar metrics collocating with the plots and aggregated to the plot level using values from the raster layers [71]. We focused on metrics often associated with AGB: canopy cover, canopy rumple, mean height, standard deviation of height, and height percentiles of returns over 2 m: 5, 10, 20, 25, 30, 40, 50, 60, 70, 75, 80, 90, and 95th percentiles [10, 65, 83, 82]. For height metrics we used all returns over 2 m above the ground, while canopy cover was calculated as the number of returns over 2 m divided by the total number of returns. Canopy rumple measures crown surface roughness, the three-dimensional measure of horizontal and vertical canopy heterogeneity, as the ratio of canopy outer surface area to ground surface area [55, 86]. For FIA, lidar-derived metrics were extracted for the true locations of FIA central subplots and these data were used in variable selection and cross-validation. In the full geospatial prediction, publicly available perturbed FIA locations were used due to limited data access (Fig. 3). For all other plots, we used zonal weighted mean values from the raster layers for variable selection, but after variable selection, we calculated the metrics for the best model from point cloud data using plot boundaries and the lidR package in R [93, 92]. This was done to get the most precise metric values for the plots, except for canopy rumple, for which we used the raster layer zonal weighted means because of the strong edge effects in this measurement [55]. We compared the raster and point cloud methods of obtaining metrics for the non-FIA plots and observed strong correlations for canopy cover (0.84) and mean height (0.91) inputs as well as minimal bias (1.83 and 0.17, respectively) and low RMSE (7.36 and 1.75, respectively). This is consistent with other research which has found similar values between point cloud and raster datasets, which result in similar predictions [71].

### Estimating biomass

We developed and tested two models to compare relative benefits and drawbacks: a null spatial model and a Bayesian geostatistical model. In order to produce AGB density at the pixel-level and mean density and total AGB estimates at the MU-level, we used the geostatistical model constructed within a Bayesian hierarchical framework to propagate uncertainty through to prediction and regional estimates [3, 28]. First we selected lidar variables to be used as inputs to the model, which we then used in a Bayesian geostatistical prediction method producing 30 m pixel-level estimates and standard deviations. To assess uncertainty compared with our field reference data and to compare the model with a null spatial model, we ran both model types in a cross-validation process. We also compared our field data with two regional AGB maps [8, 20, 46, 58]. Finally, we ran joint predictions for four management units (MUs) to calculate total and mean AGB.

#### Lidar variable selection

We assessed 17 lidar-derived metrics that may be correlated with AGB: canopy rumple, canopy cover, standard deviation height, mean height, and 13 height percentiles of returns over 2 m between 5 and 95th percentiles (see “Lidar data and preprocessing”) [10, 65, 83, 82]. To select the best combination of variables for predicting AGB, we fit ordinary least squares linear regression models using all possible two- or three-variable combinations from the 17 candidate variables. We fit models using square-root transformed AGB. We evaluated model fit using adjusted R^2^ and AIC scores. Lidar-based variable selection was conducted on all 360 plots.

In addition to lidar-derived metrics, we explored inclusion of two additional predictors: elevation and Normalized Differenced Vegetation Index (NDVI) from Landsat data during the summer of 2018. However, neither of these additional metrics added to the explanatory power of the linear model and were omitted.

#### Bayesian geostatistical prediction

In this study, we adapted the general spatial modeling framework described in Babcock et al. [3]. We assessed two models: a null spatial model and a geostatistical model that incorporates three lidar-derived metrics to predict AGB across space,both include a spatial random effect.

To assess the inherent spatial dependence structure of subalpine AGB, the null spatial model is formulated as

$$y\left( {\bf s} \right) = \beta_{0} + w\left( {\bf s} \right) + \epsilon \left( {\bf s} \right)$$

where $y({\bf s})$ is square-root transformed AGB from field measurements at location ${\bf s}$, where ${\bf s}$ is a two-dimensional coordinate vector. Only the intercept regression parameter, ${\beta}_{0}$, is estimated, and it should approximate the overall mean because there are no other regression parameters. The spatial random effect $w({\bf s})$ was modeled as a Gaussian process with a zero mean and an exponential spatial covariance function. The exponential spatial covariance function includes two parameters to be estimated: a spatial variance term (σ^2^) and a spatial decay parameter (ϕ). The error term $\epsilon ({\bf s})$ captures non-spatial variability unexplained by the spatial random effect $w({\bf s})$. More details concerning this modeling framework can be found in Babcock et al. [3] and Banerjee et al. [4].

For the geostatistical model, we predict AGB with three lidar-derived metrics. The model is written as

$$y\left( {\bf s} \right) = \beta_{0} + \beta_{1} x_{1} \left( {\bf s} \right) + \beta_{2} x_{2} \left( {\bf s} \right) +\beta_{3} x_{3} \left( {\bf s} \right) + w\left( {\bf s} \right) + \epsilon \left( {\bf s} \right)$$

where $y({\bf s})$, $w({\bf s})$, and $\epsilon ({\bf s})$ were described above. The intercept parameter, ${\beta}_{0}$, and regression slope parameters, ${\beta}_{1}$ and ${\beta}_{2}$, describe the relationship between the lidar metrics, ${x}_{1}({\bf s})$, ${x}_{2}({\bf s})$, ${x}_{3}({\bf s})$, and $y({\bf s})$. Selection of the three lidar explanatory variables, ${x}_{1}({\bf s})$, ${x}_{2}({\bf s})$, and ${x}_{3}({\bf s})$, is described in “Biomass estimation” section (variable selection).

We fit the models using the spBayes package in R [31, 33]. A Bayesian paradigm of statistical inference was employed, with vague prior distributions for all model parameters to minimize their influence on posterior inference. A Markov chain Monte Carlo (MCMC) approach was used to sample from the posterior distribution of all model parameters. The spatial parameters are the partial sill (${\sigma }^{2}$), nugget (${\tau }^{2}$), and effective range (*eff range*). We use effective range, the distance where correlation between two points falls to 0.05, because the exponential covariance function has an asymptotic range; this value is calculated as $-ln(0.05)/\phi$. Algorithms for efficient estimation of parameters are detailed in Banerjee et al. [4] and Finley et al. [32].

For the geostatistical model, MCMC-based samples from the posterior predictive distribution (PPD) of square-root AGB were obtained at all pixel locations within the study area using composition sampling. PPD samples were back-transformed (squared) to obtain AGB density PPDs in Mg/ha. Pixel-level posterior predictive medians (*Est*) and standard deviations (*SD*) were obtained by taking the median and standard deviation of AGB PPD samples for each pixel, respectively. Relative standard deviation (*RSD*) was calculated as SD/Est* 100%. Pixel-level 95% credible intervals, the range containing 95% of the posterior predictive distribution, were obtained by taking the 2.5% (lower bound) and 97.5% (upper bound) quantiles of the PPD samples at each pixel. For further details on the methodology, please refer to Babcock et al. [3].

#### Bayesian model uncertainty analysis

To assess pixel-level predictive performance, we performed a tenfold holdout cross-validation for both the null spatial and geostatistical models. We randomly assigned each plot to 10 approximately equal sized groups, and iteratively withheld each group for testing in each of 10 model runs. As in the full pixel-based estimate, we calculated median (*Est*), standard deviation (*SD*), and 95% credible interval (*CI*) for each predicted plot using back-transformed PPDs. We calculated the empirical coverage probability, the proportion of plots that have measured AGB values within the predicted 95% credible interval. We also calculated Root Mean Squared Error (RMSE) and Relative Standard Error (RSE) using back-transformed predictions from the holdout plot results and the observed field reference values.

#### Comparisons with other AGB map products

As a comparison for our Bayesian model results, we also evaluated two existing regional AGB maps from 2017: LEMMA and eMapR [8, 20, 46, 58]. For both maps, pixel values were extracted from the centroid of each plot for the 258 non-FIA plots and we calculated RMSE, RSE, and bias. We compared these values to the holdout predictions for the same plots in the tenfold cross-validation of the geostatistical model.

LEMMA, the Landscape Ecology, Modeling, Mapping & Analysis group, produces a suite of AGB metrics for California and western Oregon including live tree AGB and dead tree AGB from Landsat multi-spectral satellite imagery [8, 20]. Within each of the determined physiographic regions, of which the Sierra Nevada is one, LEMMA employs the gradient nearest neighbor (GNN) method, an imputation modeling technique that associates each satellite pixel with the FIA plot which has the most similar spectral and environmental conditions [84]. For the FIA plot training data, it uses the Component Ratio Method to estimate AGB of all live trees ≥ 2.5 cm DBH (smaller than our field data), while the dead tree AGB layer estimates AGB for snags ≥ 25 cm DBH and ≥ 2 m tall. We used two layers of 2017 maps: live tree AGB and total AGB, which we calculated by adding live tree and dead tree AGB estimates, for comparison.

The environmental monitoring, analysis, and process recognition (eMapR) lab creates AGB estimates for California, Oregon, and Washington derived from Landsat satellite imagery, FIA data, and ancillary spatial data including climate, elevation and soil type data [46, 58]. Annual composites are first created using LandTrendr’s noise-filtering, time-stabilizing algorithms, and then GNN is used where plot data are unavailable to calculate AGB. Because eMapR only estimates live tree AGB, it was compared only with live tree AGB from our field reference data.

Finally, while our Bayesian approach has clear statistical advantages by accounting for spatial structure and enabling rigorous uncertainty estimations, we also developed a non-parametric machine learning model to estimate AGB and assess the range of precision possible (Supplement A). The random forest regression model, a widely used machine learning technique, was built using the same inputs as those used for our primary modeling method. The comparison with random forest results is described in the supplemental materials (Supplement A) and discussed in the last section.

#### Management unit-level biomass estimation

We applied the Bayesian model to four management units (MUs) where complete lidar coverage was available through June 2022: Kings Canyon National Park, Yosemite National Park, Sierra National Forest, and the Lake Tahoe Basin Management Unit of the National Forest Service (Fig. 2). Full coverage was obtained from single acquisitions for Kings Canyon and Lake Tahoe Basin, and from multiple acquisitions for Sierra National Forest and Yosemite. AGB density PPDs across MU’s were generated via MCMC-sample based integration of the pixel-level PPDs. Once MU-level PPDs are obtained, they can be summarized similarly to pixel-level PPDs (e.g., calculate PPD medians, standard deviations and credible intervals). Details about how to obtain MU-level estimates using Bayesian spatial models can be found in Babcock et al. [3] and references therein.

We report the aggregated estimates of AGB and uncertainty over each MU in terms of total values and mean density of AGB. We compare these values to summarized totals and means for LEMMA and for mean and SD of our lidar predictor variables.

## Results

### Lidar variable selection

**Fig. 4:**
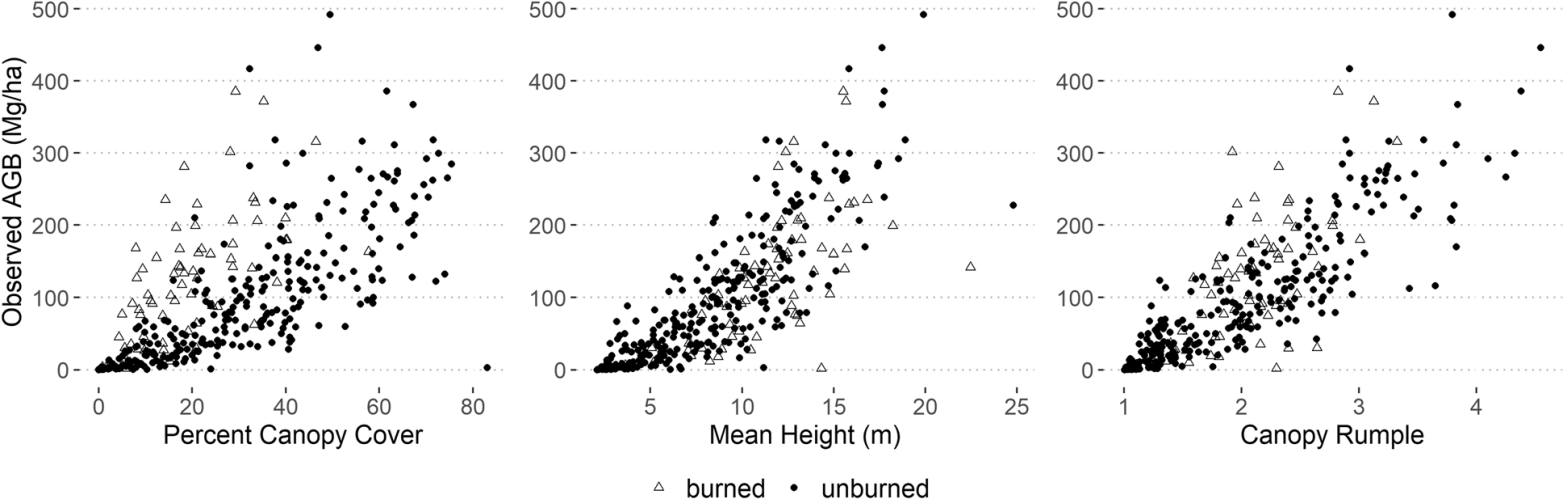
Aboveground biomass (Mg/ha) increases with percent canopy cover (left), mean height (center), and canopy rumple (right). Burned plots tend to have lower canopy cover along with lower correlation coefficients than the unburned plots

The best multiple linear model predicting total AGB included canopy rumple, canopy cover, and mean height (adjusted R^2^ = 0.79). These variables showed moderate to high collinearity (correlation coefficient = 0.63 canopy cover:mean height; 0.77 canopy cover:canopy rumple; 0.85 canopy rumple:mean height). These three metrics each have good correlation with AGB, and especially with square root transformed AGB, the model response variable: 0.74 (canopy cover), 0.84 (mean height), and 0.85 (canopy rumple) (Fig. 4). Correlations with square root transformed AGB were higher for unburned plots than burned, with values for canopy cover, mean height, and canopy rumple, respectively, of 0.66, 0.71, and 0.68 for burned plots and 0.83, 0.86, and 0.88 for unburned plots.

### Biomass estimation

#### Bayesian geostatistical prediction

**Fig. 5:**
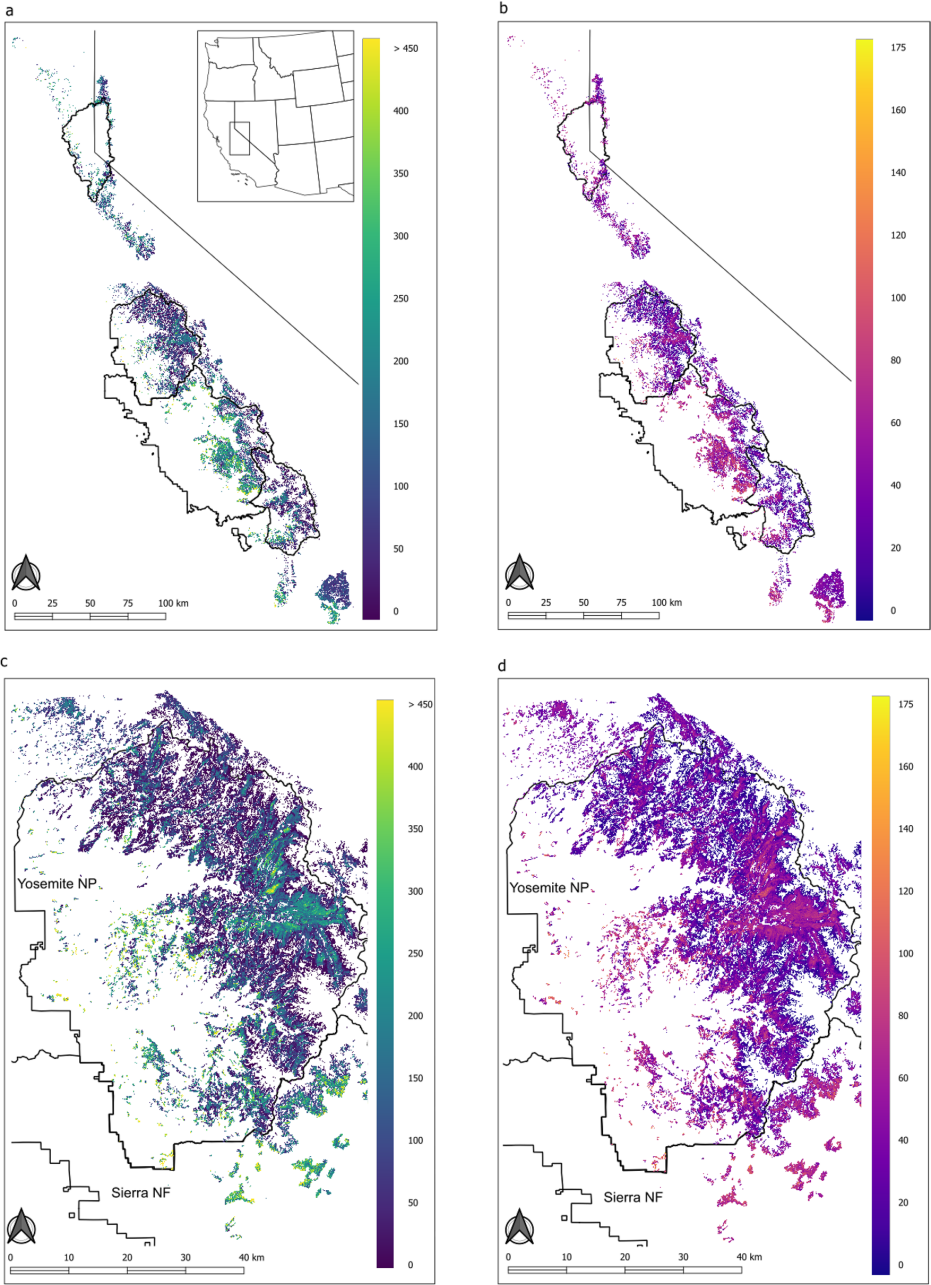
Output maps from the full pixel-based model showing the estimate (**a**, **c**) and standard deviation (**b**, **d**) for the full region (**a**, **b**) and a zoomed in area over Yosemite National Park, California (**c**, **d**)

Since the null spatial model contains no explanatory variables, the partial sill (${\sigma }^{2}$), nugget (${\tau }^{2}$), and effective range (*eff range*) parameter estimates can be examined to assess the inherent spatial dependence structure of subalpine AGB. We see from Table 2 that ${\sigma }^{2}$ is substantially higher than that ${\tau }^{2}$. The nugget-to-sill ratio $({\tau }^{2}/[{\tau }^{2}+{\sigma }^{2}$]) is 0.04, indicating an extreme amount of spatial dependence in subalpine AGB. Moving to the geostatistical model, ${\sigma }^{2}$ reduces from 20.48 to 4.20, suggesting that a substantial amount of the spatial dependence in AGB is explained by the lidar explanatory variables. However, not all spatial variability in AGB is explained by the lidar variables (${\sigma }^{2}$ is not zero), meaning that, in order to conduct valid model-based inference, the spatial random effect in the geostatistical model needs to remain. The pixel-based geostatistical model yielded smooth maps of AGB predictions and SD, with higher estimates often associated with higher SD (Fig. 5). The mean AGB estimate for subalpine pixels was 133.4 Mg/ha with a SD of 119.4 Mg/ha, reinforcing that AGB is very heterogeneous in the region. Most pixel values were low, with 95% under 367 Mg/ha (Figure S1).

**Table 2:** Parameter posterior quantile summaries for the null spatial and geostatistical models

	Null spatial	Geostatistical
Parameter posterior quantile summaries 50% (2.5%, 97.5%)		
β_0*y*_	8.91 (8.35, 9.58)	− 1.20 (− 1.88, − 0.43)
τ^2^	0.83 (0.21, 13.68)	0.72 (0.20, 3.22)
σ^2^	22.48 (9.48, 26.79)	4.20 (1.59, 5.29)
Eff range (km)	0.32 (0.06, 0.76)	0.12 (0.07, 0.37)

An interactive map of the results is available through a Google Earth Engine app at https://swinsem.users.earthengine.app/view/subalpine-forest-biomass. AGB predictions and SD for subalpine forests are visualized along with high resolution imagery from the National Agriculture Imagery Program (NAIP), and the value of AGB can be extracted at each subalpine forest pixel.

#### Bayesian model uncertainty analysis

In the tenfold cross validation, the null spatial model showed high levels of error in terms of RMSE, RSE, and R^2^, exemplified by the R^2^ value 0.01 (Table 3). This is not surprising given that AGB heterogeneity is high in this region and no explanatory variables are included in the model. The null spatial model serves as a benchmark to gauge the performance of the lidar explanatory variables. The null spatial model predictions were centered around the mean of the AGB field measurements used to train the model. The mean credible interval width was 326 Mg/ha indicating a high degree of prediction uncertainty. The empirical 95% coverage probability was 93.61%, suggesting that model-based assumptions were not seriously violated.

**Table 3:** Uncertainty measurements from the tenfold cross-validations for the null spatial and geostatistical models

	Null spatial	Geostatistical
*RMSE* (Mg/ha)	96.38	49.06
*RSE*	105.76%	27.41%
*R*^*2*^	0.01	0.73
Bias (Mg/ha)	− 23.95	− 4.64
95% coverage probability	93.61%	94.72%
Mean 95% CI width (Mg/ha)	326.0	152.7

We report five measures of model uncertainty: root mean square error (RMSE), relative squared error (RSE), R squared (R^2^), bias [mean(predicted − observed], 95% coverage probability (the percent of measured plot values that fall within the calculated 95% credible interval (null and spatial), and the mean width of the 95% credible interval. Note that while the null model has good coverage probability due to very wide credible intervals, the RMSE, RSE, and R^2^ values are poor

**Fig. 6:**
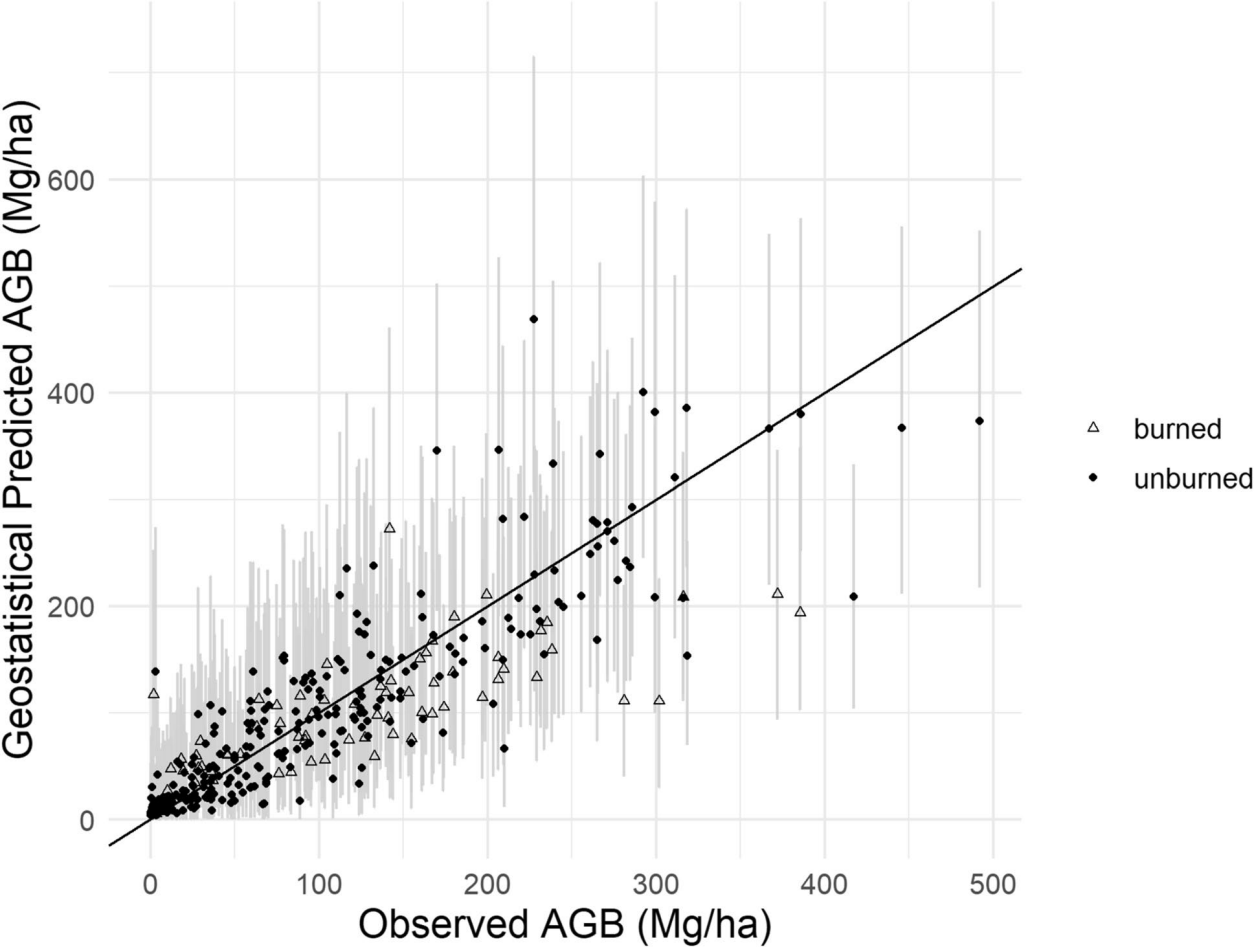
Cross-validation results comparing field-measured AGB with the model predicted AGB for the geostatistical model. Vertical gray lines show the 95% credible interval, which is the range containing the central 95% of predicted values for each plot

For the geostatistical model, the tenfold cross validation yielded a RMSE of 49.2 Mg/ha, RSE of 27.56%, and bias of −4.64 Mg/ha (Table 3), indicating a dramatic increase in prediction accuracy and precision over the null spatial model. Mean credible interval width was 152.2 Mg/ha. Empirical coverage probability was 94.72%, as 341 of 360 had field-measured AGB within the calculated 95% credible interval (Fig. 6).

Of the 19 plots outside the 95% coverage probability, the model underestimated 11 plots and overestimated 8 plots. Of the plots outside the credible interval, where the field-measured value was not contained in the central 95% of the prediction distribution, 4 were FIA plots, 7 were post-fire plots, and 8 were R5 whitebark plots; no NPS I&M plots were outside its credible interval. Five of the plots outside the credible interval had burned in recent fires, four of which were underestimated, likely because the canopy cover measurement would be relatively low when there is a high proportion of dead trees.

#### Comparisons with other AGB map products

**Fig. 7:**
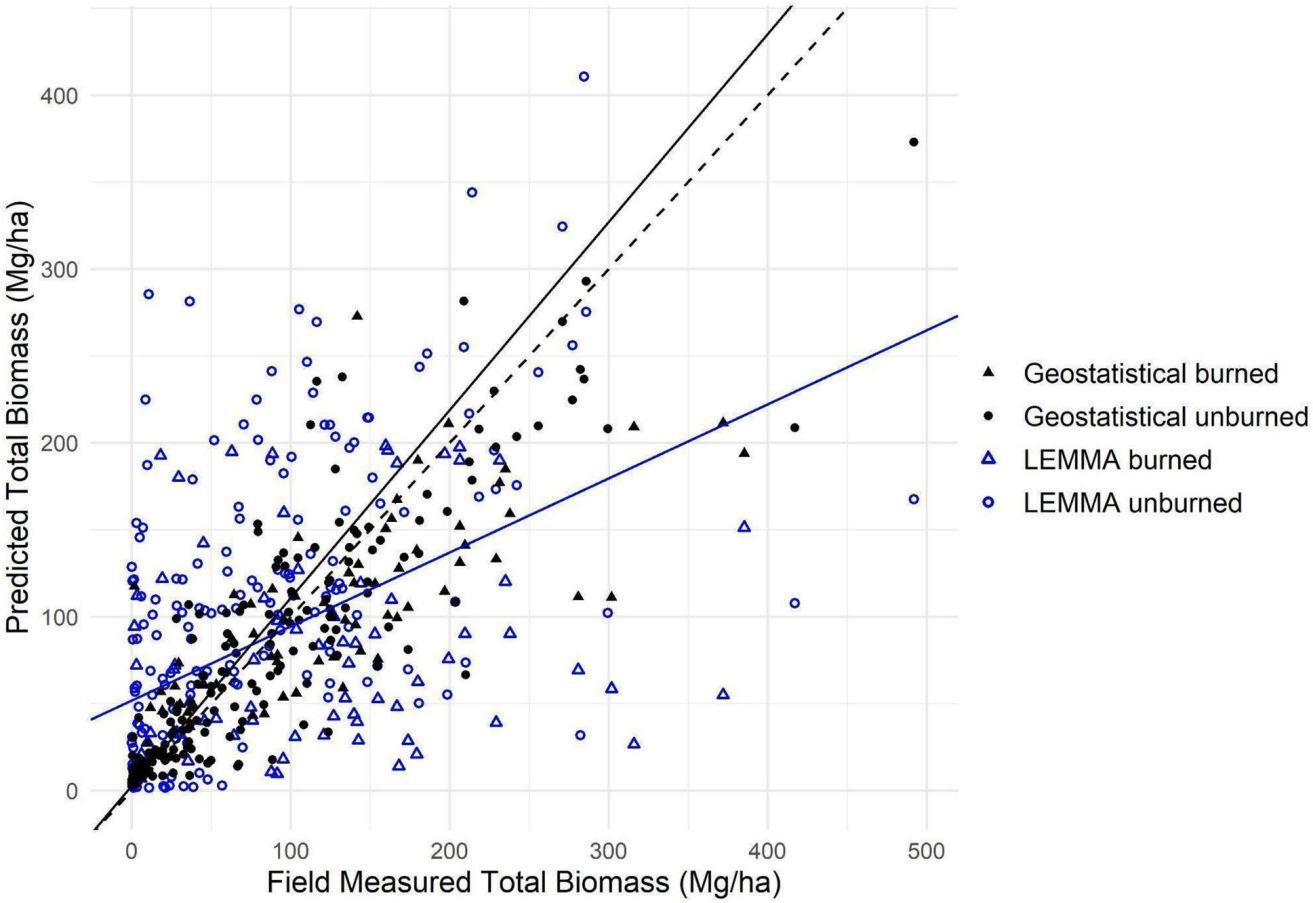
Comparison of our model estimate and total AGB from LEMMA (live tree + dead tree estimates) with the observed field reference values. Our estimate’s slope (1.08) and intercept (2.61) are shown with the solid black line, while LEMMA’s slope (0.43) and intercept (51.84) are shown with the blue line. On average, estimates from our model are closer to the field measured biomass represented by the dashed 1:1 line

When evaluated against 258 non-FIA plots, error metrics of RMSE, RSE, and bias were lower for our model than the two regional models we used for comparison, LEMMA and eMapR (Table 4). We compared total AGB from our model with live tree and total AGB (the sum of live and dead tree AGB) for LEMMA, while we used only live tree AGB for comparison with eMapR. LEMMA predictions often overestimated for plots with lower AGB and underestimated plots with higher AGB (slope = 0.43, intercept = 51.84) (Fig. 7). However, our model estimations were much closer to the observed values across the whole gradient of AGB (slope = 1.08, intercept = 2.61). The 258 plots were used in training and testing the geostatistical model in a tenfold cross validation, but were independent of the LEMMA and eMapR pipelines.

**Table 4:** Error metrics for our geostatistical model, LEMMA live and total, and eMapR, for 258 non-FIA plots, where exact locations are available

Dataset	RMSE (Mg/ha)	RSE (%)	Bias (Mg/ha)
This Study (total AGB)	46.4	28.8	− 4.64
LEMMA (live AGB)	84.1	108.3	20.95
LEMMA (total AGB)	93.1	106.5	7.50
eMapR (live AGB)	66.6	73.9	19.54

The random forest model yielded comparable results to the geostatistical model, with similar values for RMSE, RSE, R^2^, coverage probability, and 95% confidence interval width (Table S.A1). There appears to be some leveling off of predicted values at high observed AGB measures, which could indicate underprediction in the random forest model (Fig. S.A1). Additionally, a map of standard deviation from the random forest model shows less spatial coherence compared with the geostatistical model (Figure S.A2).

#### Management unit-level biomass estimation

We found clear differences in AGB density of subalpine forested areas across different MUs (Table 5). Kings Canyon National Park, the southernmost region, had the lowest AGB density (92.4 Mg/ha [CI 87.9, 96.5]), less than half the density of forests in Sierra National Forest (199.2 [CI 188.4, 208.3] Mg/ha). Yosemite National Park’s subalpine forest AGB was also relatively low (106.5 Mg/ha [CI 101.7, 111.7]), while Tahoe Basin had intermediate AGB density (165.4 Mg/ha [157.2, 173.6]). Total AGB is relative to the area of subalpine forest, so it follows that the smallest region (Tahoe Basin) had the least area and least total AGB, while Sierra National Forest had the highest total AGB estimate, given the highest AGB density and its relatively large area.

**Table 5:** Total AGB estimates and predictive precision for each of the four management units analyzed

	Kings Canyon National Park	Sierra National Forest	Tahoe basin management unit (USFS)	Yosemite National Park
Area (ha)	63,099	105,463	13,526	107,735
Total AGB (Tg)				
Est	5.8 (5.6, 6.1)	21.0 (19.9, 22.0)	2.2 (2.1, 2.3)	11.5 (11.0, 12.0)
SD	0.14	0.58	0.06	0.28
RSD	2.3%	2.8%	2.7%	2.4%
Mean AGB (Mg/ha)				
Est	92.4 (87.9, 96.5)	199.2 (188.4, 208.3)	165.4 (157.2, 173.6)	106.5 (101.7, 111.7)
SD	2.2	5.5	4.4	2.6
RSD	2.3%	2.8%	2.7%	2.4%
LEMMA Total AGB (Tg)				
Est	6.1	20.4	1.8	12.9
LEMMA Mean AGB (Mg/ha)				
Est	93.2	192.4	160.6	116.8

Estimate = median estimated AGB (95% credible interval in parentheses), SD = posterior predictive standard deviation for AGB, and RSD = relative standard deviation for AGB estimate (SD/Est * 100%). The bottom two rows show LEMMA values for subalpine forests, summarized to the same management units

Values for LEMMA had very similar total and mean estimate values when summarized to MU, however credible intervals, SD, and RSD could not be calculated (Table 5). When summarized to these large areas, errors in estimation are largely averaged out. However, some of the LEMMA values are outside of our model’s credible intervals: Tahoe Basin MU’s total AGB is lower than our credible interval, and Yosemite National Park’s total and mean AGB estimates are above our credible intervals (Table 5). Because eMapR only produced live tree AGB, we did not compare it with our model outputs.

Further analysis showed that, compared with the Sierra National Forest and Tahoe Basin MU, Kings Canyon and Yosemite had much lower mean canopy cover (around 28%) and mean height (7.8 ± 4.4 m and 8.9 ± 4.4 m) (Table 6), which likely explained their relatively lower AGB density. In contrast, mean canopy cover was highest in Sierra National Forest (48.4%) and trees were taller (mean 12.1 ± 4.6 m), resulting in the highest mean AGB density.

**Table 6:** Management unit mean and standard deviation (in parentheses) for the lidar predictor variables, percent canopy cover, canopy rumple, and mean height

	Kings Canyon National Park	Sierra National Forest	Tahoe Basin Management Unit (USFS)	Yosemite National Park
% canopy cover	28.0 (19.7)	48.4 (19.5)	44.5 (19.0)	27.6 (18.7)
Canopy rumple	1.8 (0.9)	2.7 (1.0)	2.6 (0.8)	2.1 (0.9)
Mean height	7.8 (4.4)	12.1 (4.6)	10.9 (4.3)	8.9 (4.4)

The areas with higher values in all three also had higher AGB values

## Discussion

Although AGB has been modeled regionally, nationally, and globally with reasonable overall accuracy [26, 49, 58], the aggregate nature of these broad efforts can lead to lower accuracy in less prevalent forest types, which are overshadowed by the characteristics of dominant forest types. We quantified accuracy, precision, and model calibration in subalpine forests of the Sierra Nevada with the first ever application of a Bayesian geostatistical model and for two regional AGB maps.

We modeled AGB for subalpine forests using a large collection of training data and aerial lidar data in a Bayesian geostatistical model, enabling us to assess AGB estimates and uncertainty at fine and broad scales. Compared with other regional AGB mapping efforts, our RMSE and RSE error measures were notably lower, indicating that our locally trained model provided maps had better agreement with field reference data.

### Biomass estimation

#### Variable selection

The variables that led to the best model fit, canopy cover, canopy rumple, and mean height, provide information about the horizontal and vertical distribution of AGB (Fig. 4). They all exhibit good correlation with AGB and only moderate correlation with each other, providing a solid foundation for the prediction. Canopy cover tended to be lower for burned plots with the same AGB measurements, which aligns with previous research showing decreased canopy cover after fire, including low severity [56]. While we included a high proportion of burned plots and so might have expected that including NDVI could improve the model, we measured NDVI in 2018, 4–17 years after fire, so any relevant signal of the burn may have been less apparent [59].

We estimated total standing woody AGB, including snags, in this study because subalpine forests have very slow decomposition. We did not include coarse woody debris, litter, duff, shrub, or herbaceous layers due to the relatively lower values and the difficulty of observing those aspects of data from any kind of remote sensing; these were also not included in our comparison datasets [8, 58]. Coarse woody debris is a persistent source of AGB similar to snags in these systems, however, and research showed that in subalpine forests in the Rocky Mountains, it had an average turnover time of 580 ± 180 years [60]. Total standing AGB has also been calculated in other studies [68], and we believe it is more relevant for subalpine forests than live AGB due to the long residence time of dead wood.

#### Models and uncertainty analyses

The geostatistical model had relatively low values of RMSE and RSE in the cross-validation, indicating good model fit. However, the 95% credible intervals, which encapsulate the distribution of the center 95% of estimates, were wide (Table 3). Roughly 95% of credible intervals included the measured AGB value, indicating appropriate credible interval width and adequate model-based uncertainty estimation. Burned plots were more likely to be outside of the credible interval (Fig. 6), which may be related to the lower canopy cover measurements for similar AGB levels due to dead tree presence (Fig. 4). AGB studies that have focused on subalpine forests in other regions have obtained lower RMSE than our study, however they also have lower mean AGB, more similar forest types modeled (i.e. fewer species types or narrower elevational ranges in the studies), and significantly smaller regions and plot sample sizes [22, 97]. This study is the first to model subalpine forests across such a broad, diverse subalpine region and to include mapped uncertainty and estimated total AGB in management areas.

The null model shows the amount of spatial autocorrelation in the field data. The geostatistical model, in comparison, shows the remaining unexplained variability absorbed by the spatial random effect. Lidar variables effectively explain much, but not all, of the variability (Table 2), demonstrating the utility of using the geostatistical model with the spatial random effect.

#### Comparisons with other AGB map products

Our AGB estimates had higher accuracy in our subalpine forests than the two regional maps we tested, both of which were developed for large areas across many forest types (Table 4, Fig. 7). This would be expected due to the increased plot density, the use of lidar rather than optical imagery, and the focus on developing a model specific to a narrow set of forest types. Lower-elevation, higher-AGB plots dominate standard training datasets such as FIA, which could be why LEMMA and eMapR tend to overestimate subalpine AGB at the low values. At the high biomass end, underestimates of AGB are likely due to saturation from satellite imagery [116]. Low density forests with bright rocky substrates may also obscure signals from vegetation in optical imagery. Additionally, while GNN is a powerful method, the limited number of FIA plots used for training in this particularly heterogeneous region may introduce bias and reduce the precision of the resulting AGB maps. While FIA is a valuable dataset and one that we included here, including multiple plot data sources increased the breadth of forest structures represented to train our model.

When aggregated to MUs, the underestimation of LEMMA at higher biomass areas may cancel out the overestimation at lower biomass (Fig. 7). With our plot data showing lower RMSE and RSE values compared with LEMMA (Table 4), we posit that our MU credible intervals are more likely to contain the true values. This would indicate that LEMMA overestimates subalpine AGB total and density in Yosemite, and underestimates total subalpine AGB in the Tahoe Basin MU.

Given that general regional models of AGB don’t perform as well within these subalpine regions, as we demonstrated with comparisons to LEMMA and eMapR, we see the need for forest type specific models to measure current carbon and track changes. While regional models have an important role for estimating regional AGB, accuracy should be assessed in less common forest types to ensure that local applications and management decisions are sound. This mirrors research in a different specialized forest type, mangroves, which found that local calibration was important for representing area-wide total AGB and that forests with low to moderate AGB density could see the biggest model improvements [102]. They found the highest relative standard deviation in shorter forests and noted that capturing variability in canopy structure was more important than absolute height accuracy [102], which mirrors our finding of canopy rumple as an important predictor.

As a widely used non-parametric machine learning approach, the random forest regression model yielded comparable results to the geostatistical model, however it exhibited certain limitations (Supplement A). Specifically, the random forest tended to underpredict at high observed AGB measures, indicating more saturation in the random forest model than in the geostatistical model (Fig. S.A1). It is common for non-parametric methods to not extrapolate beyond the range of training data; perhaps the scarcity of high AGB plots in the training dataset limited the random forest model’s capacity to capture the true range of AGB, similar to findings of random forest classification with imbalanced class sizes [17]. Additionally, the random forest-derived map of standard deviation (Figure S.A2) exhibited reduced spatial coherence compared to the geostatistical model, giving a more “noisy” appearance. This increased variability could indicate lower interpretability of variance measures, especially given that our calculations of standard deviation (from the cross-validation holdout trees) and confidence intervals (approximated as SD*1.96) relied on Gaussian assumptions which do not apply to random forest models. Future work using non-parametric models should explore alternative methods for uncertainty quantification.

#### Regional differences in management unit-level biomass estimation

Estimation and uncertainty assessment at the MU level provides managers and policymakers with improved carbon accounting numbers. While summing up individual pixels can be done within a region, the joint prediction within the Bayesian geostatistical framework gives rigorous uncertainty estimations and credible intervals. AGB density varied between our MUs, with roughly double the AGB density in Sierra National Forest as we found in Kings Canyon and Yosemite National Parks (Table 5), consistent with variation in lidar predictors between the MUs (Table 6). The two national parks are mostly at higher elevations than the USFS regions, and elevation was negatively correlated with AGB. Additionally, the national parks have rockier soil underlain principally by granitoid batholiths, while the national forest regions have notable extents of volcanic and metasedimentary rocks in addition to granitoid rocks [5]. These elevational and soil factors could explain some of these regional differences in both lidar metrics and AGB density.

#### Limitations/sources of error

For accurate modeling using plot data, it is essential to have a sufficient number of plots to represent the variety of vegetation conditions, including a range of disturbance histories [80, 112]. Including four plot datasets, one of which focused on post-fire forests, allowed us to improve our estimates and our uncertainty calculations especially in burned areas. Heterogeneity and uniformity of forest development impact uncertainty in AGB estimation: in one study, more structurally diverse mountainous forested sites had R^2^ = 0.77 while conifer plantations had R^2^ = 0.94 [12]. Non-plantation Douglas-fir forests, while still heterogeneous especially due to disturbances, have strong patterns of development stages [35], which may help translate to better estimation of AGB. Subalpine forests, however, have many different species and growth patterns across short spatial scales [34, 90], challenging AGB mapping.

Error is involved at many steps of AGB mapping, and while the chosen Bayesian method does propagate some error (e.g. model parameter error and lack-of-fit to model) through the steps, additional errors common to many AGB mapping efforts are not delineated. Imprecision in field measurements of trees is possible especially for height measurements when a clear view of the tree top or bottom is not possible. We also likely had errors from differing plot protocols and sampling teams, including possible bias due to different plot selection methods. Other research has found that smaller plots lead to predictions with lower accuracy, lower precision, and higher bias [36, 37, 104] and that absolute model error declines with increasing plot size [116]. GPS positioning inaccuracies are more pronounced in areas of steep topography and narrow canyons where subalpine forests often grow, and we acknowledge potential errors due to location mismatches between our field and lidar data. Inaccuracy in GPS positioning exacerbates errors in extracted lidar values for smaller plots [36]. Smaller plots also have greater edge effects due to tree crowns along the plot boundary unintentionally falling in or out of the plot [36], which could be especially important in heterogeneous regions with small forest patch sizes. Accuracy assessments using field plots that are smaller than the map resolution could underestimate map accuracy, leading to overly pessimistic error calculations–this would have applied to our assessment of LEMMA and eMapR as well as our own model [11]. This may explain the 100% coverage probability of NPS I&M plots (the largest size plot). Future research that uses larger plots would be a valuable addition to improve AGB estimates.

Error in allometry comes from multiple sources, including the limited number of trees used to develop each species’ base biomass equation and the fact that allometric studies are conducted in specific sites that have site-specific morphology and growth patterns which may differ from the site at hand [68]. Some tree species may have allometry from other mountain ranges like the Rocky Mountains or the Cascades, and at some diameter ranges they may have a more general equation, such as an equation for *Pinus *spp*.* rather than for the particular species. We used the Component Ratio Method (CRM) because it is the standard equation used by FIA and therefore for maps such as LEMMA and eMapR, however the choice of equation can lead to different estimates and different model accuracy [115]. We assessed plot biomass with and without trees 2.5–7.5 cm DBH and found these small trees contributed minimal AGB. This indicates that using our field data with trees > 7.6 cm DBH to assess LEMMA and eMapR, which mapped AGB for trees > 2.5 cm DBH, should not introduce significant bias.

In our lidar processing, some values remained that were unreasonable. We removed pixels with mean height over 40 m and 95th percentile height over 64 m because these higher values were definitely unreasonable for trees in this forest type, however some pixels retained could still have faulty measurements possibly caused by steep terrain changes. Plot sampling tends to under-sample rare, very large trees [103], so we did not filter based on the maximum size found in our plots in order to prevent filtering out legitimately large tree measurements. While 99% of our pixel values had estimates below 509.5 Mg/ha, the model estimated 0.1% of pixels between 745.5 and 1330.9 Mg/ha (Figure S1). Most of these outliers are likely due to inaccurate lidar height measurements, such as overestimation caused by returns from cliff faces and rocky outcrops. Misinterpretation of topographic features during lidar processing can include misclassification of ground and canopy points on steep slopes, which could, for example, result in a 30 m pixel with an unreasonable dominant height due to the presence of a cliff. Because the filtered pixels were from incorrect measurements rather than correctly measured very tall trees, and LEMMA and eMapR use optical satellite imagery rather than lidar, we do not believe the filtering would influence the comparison of the geostatistical model with the two regional maps.

All of our lidar datasets were collected as snow-free ground reference data and they varied in pulse density from 2–27 pulse/m^2^ and collection year from 2014–2021 (Table S1). We were not able to test whether point density was correlated with accuracy. However, research has shown that point densities above 1 pulse/m^2^ still have high accuracy for measurements of height and canopy cover, and that AGB can be calculated well for our range of pulse densities [51, 105]. The discrepancies between field data collection year (2013–2021) and lidar collection (2014–2021) could have also led to lower accuracy in our prediction. All fires from our post-fire dataset burned before the lidar collections, however tree mortality or snag fall between field and lidar collection could have led to bias depending on which was measured first. Our assessment of LEMMA and eMapR could also be biased because these products were predicted for 2017 but were assessed with field data collected before and after. Because field and lidar data are so limited in this region, these discrepancies were unavoidable for this project, however future projects should try to align the collection years better.

This study focused solely on standing woody aboveground AGB from live and dead trees. We did not include shrubs due to the lack of allometry in the literature and the difficulty measuring shrub cover from lidar [54]. This included the exclusion of Krummholz whitebark pine because we did not find a method for calculating AGB. However, we don’t think this is a problem for the model interpretation because shrub AGB accounts for a relatively small proportion of AGB in the subalpine due to generally low cover and low stature of shrubs [34], the reduced post-fire shrub response in subalpine forests compared with lower-elevation forests, and that even in lower-elevation forests, shrubs account for a small amount of biomass in late-successional forests (1%) and 40 years after stand-replacing fire (6%) [40, 67].

Despite the improvements in our model, we still had wide credible intervals and sizable standard deviations and relative standard deviations at the pixel level. In the Bayesian geostatistical method, pixel level predictions often have large errors, while joint predictions over larger areas have relatively low error [3]. Our results follow this pattern, as credible intervals are much narrower and relative SD is small in our MU estimates. In a region with such high heterogeneity and topography that challenges remote sensing signals, errors may always be higher in this region than in flatter and more homogeneous regions [12].

### Synthesis

The methods explored here have the potential to improve our estimation of forest AGB in and beyond the subalpine region and demonstrate the advantage of ecosystem-specific modeling for underrepresented forest types. Statistically rigorous uncertainty metrics alongside AGB estimates are important contributions to carbon trading markets, conservation, and management. Subalpine forests face increasing threats, punctuated by 2021, when two fires burned over the Sierra Nevada crest, and 2022, when whitebark pine was listed as “Threatened” under the US Endangered Species Act primarily due to the spread of white pine blister rust, along with altered climate (warmer temperatures and less snow), mountain pine beetle, and altered fire regimes [107]. Understanding the contribution of subalpine forests to the overall carbon storage in the region is an important baseline in order to understand our current and future forest management decisions.

## Conclusion

Our study produced the most precise and accurate aboveground biomass estimates to date for Sierra Nevada subalpine forests. To capture the variability of forest structure in these forests, we combined four field datasets with lidar data and employed a Bayesian geostatistical method that has been highly successful in other regions [3, 28]. Subalpine forests of the Sierra Nevada have highly varied forest structure, but increased data availability and improvements in modeling methods resulted in a model with rigorous uncertainty assessment and improved performance compared with two regional models. Our cross-validation process gave an empirical 95% coverage probability of 94%, indicating adequate model-based uncertainty estimation. RMSE, RSE, and bias for our geostatistical model were much lower compared with two regional models, both of which use optical satellite imagery for monitoring change over time. This method provides estimates of baseline AGB in subalpine forests, because lidar is not currently available at full coverage or with repeat visits for tracking change over time. Within the subalpine and beyond, application of these methods would improve our understanding of AGB estimates and uncertainties across scales from the pixel to the regional level. This research also demonstrates that focusing on a distinct, specialized forest type can improve AGB estimation over broad regional models, and suggests that modeling can be improved in other heterogeneous regions, including subalpine forests worldwide, with similar methods. Accurately quantifying forest AGB is essential in the face of accelerating changes in forest structure due to climate change, fire, and drought. Forests play a critical role in our environment and economy, and understanding their carbon dynamics is key to managing them sustainably.

## Supplementary Information

Supplementary Material 1.

## Data Availability

Biomass maps (estimate and standard deviation) are available on Earth Engine and upon request. Some input data are available upon request, however, true locations of FIA plot data are not publicly available and cannot be provided.

## Abbreviations

AGB: Aboveground biomass
ASO: Airborne Snow Observatory
CALVEG: Classification and Assessment with Landsat of Visible Ecological Groupings
DBH: Diameter at breast height
eMapR: Environmental Monitoring, Analysis, and Process Recognition
FIA: Forest inventory and analysis
GNN: Gradient nearest neighbor
GPS: Global positioning system
LEMMA: Landscape Ecology, Modeling, Mapping & Analysis
MU: Management unit
NDVI: Normalized differenced vegetation index
NPS: National Park Service
PPD: Posterior predictive distribution
R2: Coefficient of determination
RMSE: Root mean squared error
RSD: Relative standard deviation
RSE: Relative standard error
SD: Standard deviation
USDA: United States Department of Agriculture
USFS: United States Forest Service

## References

[1] Abdalati W, Zwally HJ, Bindschadler R, Csatho B, Farrell SL, Fricker HA, Harding D, Kwok R, Lefsky M, Markus T, Marshak A, Neumann T, Palm S, Schutz B, Smith B, Spinhirne J, Webb C. The ICESat-2 laser altimetry mission. Proc IEEE. 2010;98:735–51. 10.1109/JPROC.2009.2034765.

[2] Alizadeh MR, Abatzoglou JT, Luce CH, Adamowski JF, Farid A, Sadegh M. Warming enabled upslope advance in western US forest fires. Proc Natl Acad Sci. 2021;118: e2009717118. 10.1073/pnas.2009717118. 34031237 10.1073/pnas.2009717118 PMC8179236

[3] Babcock C, Finley AO, Andersen HE, Pattison R, Cook BD, Morton DC, Alonzo M, Nelson R, Gregoire T, Ene L, Gobakken T, Næsset E. Geostatistical estimation of forest biomass in interior Alaska combining Landsat-derived tree cover, sampled airborne lidar and field observations. Remote Sens Environ. 2018;212:212–30. 10.1016/j.rse.2018.04.044.

[4] Banerjee S, Carlin BP, Gelfand AE. Hierarchical modeling and analysis for spatial data. In: Monographs on statistics and applied probability. 2nd ed. CRC Press; 2015.

[5] Bateman PC. Pre-Tertiary bedrock geologic map of the Mariposa 1 degree by 2 degrees Quadrangle, Sierra Nevada, California; Nevada. US Geological Survey, Miscellaneous Investigations Series, Map I-1960; 1992.

[6] Bebi P, Seidl R, Motta R, Fuhr M, Firm D, Krumm F, Conedera M, Ginzler C, Wohlgemuth T, Kulakowski D. Changes of forest cover and disturbance regimes in the mountain forests of the Alps. For Ecol Manag Ecol Mountain Forest Ecosyst Europe. 2017;388:43–56. 10.1016/j.foreco.2016.10.028. 10.1016/j.foreco.2016.10.028 PMC5572777 28860675

[7] Bechtold WA, Patterson PL. The enhanced forest inventory and analysis program—national sampling design and estimation procedures, GTR SRS-80. USDA Forest Service Southern Research Station, Asheville, NC; 2005.

[8] Bell DM, Acker SA, Gregory MJ, Davis RJ, Garcia BA. Quantifying regional trends in large live tree and snag availability in support of forest management. For Ecol Manage. 2021;479: 118554. 10.1016/j.foreco.2020.118554.

[9] Bell DM, Bradford JB, Lauenroth WK. Mountain landscapes offer few opportunities for high-elevation tree species migration. Glob Change Biol. 2014;20:1441–51. 10.1111/gcb.12504. 10.1111/gcb.12504 24353188

[10] Bell DM, Gregory MJ, Kane V, Kane J, Kennedy RE, Roberts HM, Yang Z. Multiscale divergence between Landsat- and lidar-based biomass mapping is related to regional variation in canopy cover and composition 07 Agricultural and Veterinary Sciences 0705 Forestry Sciences 09 Engineering 0909 Geomatic Engineering. Carbon Balance Manag. 2018. 10.1186/s13021-018-0104-6. 10.1186/s13021-018-0104-6 PMC6138055 30218413

[11] Bell DM, Gregory MJ, Roberts HM, Davis RJ, Ohmann JL. How sampling and scale limit accuracy assessment of vegetation maps: a comment on Loehle et al. (2015). Forest Ecol Manag. 2015;358:361–4. 10.1016/j.foreco.2015.07.017.

[12] Bouvier M, Durrieu S, Fournier RA, Renaud J-P. Generalizing predictive models of forest inventory attributes using an area-based approach with airborne LiDAR data. Remote Sens Environ. 2015;156:322–34. 10.1016/j.rse.2014.10.004.

[13] Breidenbach J, McRoberts RE, Astrup R. Empirical coverage of model-based variance estimators for remote sensing assisted estimation of stand-level timber volume. Remote Sens Environ. 2016;173:274–81. 10.1016/j.rse.2015.07.026. 28148972 10.1016/j.rse.2015.07.026 PMC5268351

[14] Breiman L. Random Forests. Eur J Math. 2001;45:5–32. 10.1017/CBO9781107415324.004.

[15] Brodie EG, Stewart JAE, Winsemius S, Miller JED, Latimer AM, Safford HD. Wildfire facilitates upslope advance in a shade-intolerant but not a shade-tolerant conifer. Ecol Appl. 2023;33: e2888. 10.1002/eap.2888. 37212209 10.1002/eap.2888

[16] Brown JK. Weight and density of crowns of Rocky Mountain conifers. Res. Pap. INT-RP-197. Ogden, UT: U.S. Department of Agriculture, Forest Service, Intermountain Forest and Range Experiment Station. 56 p. 197; 1978.

[17] Chen C, Liaw A, Breiman L. Using random forest to learn unbalanced data (Technical Report 666). Statistics Department: University of California at Berkeley; 2004.

[18] Cressie N. Statistics for spatial data. Incorporated: John Wiley & Sons; 1993.

[19] Das AJ, Stephenson NL. Assessment of climatic change, in: Sydoriak, C., Panek, J.A., Battles, J.J., Nydick, K.R. (Eds.), A Natural Resource Condition Assessment for Sequoia and Kings Canyon National Parks. NPS/SEKI/NRR-2013/665, Fort Collins, CO: U.S. Department of the Interior, National Park Service; 2013. p. 243–50.

[20] Davis RJ, Bell DM, Gregory MJ, Yang Z, Gray AN, Healey SP, Stratton AE. Northwest Forest Plan—the first 25 years (1994–2018): status and trends of late-successional and old-growth forests. Gen. Tech. Rep. PNW-GTR-1004. Portland, OR: U.S. Department of Agriculture, Forest Service, Pacific Northwest Research Station; 2022. 82 p. 1004. 10.2737/PNW-GTR-1004

[21] Dolanc CR, Thorne JH, Safford HD. Widespread shifts in the demographic structure of subalpine forests in the Sierra Nevada, California, 1934 to 2007. Glob Ecol Biogeogr. 2013;22:264–76. 10.1111/j.1466-8238.2011.00748.x.

[22] Du J, He Z, Chen L, Yang J, Zhu X, Zhao W. Integrating lidar with Landsat data for subalpine temperate forest aboveground carbon estimation. Int J Remote Sens. 2015;36:5767–89. 10.1080/01431161.2015.1101651.

[23] Dubayah R, Blair JB, Goetz S, Fatoyinbo L, Hansen M, Healey S, Hofton M, Hurtt G, Kellner J, Luthcke S, Armston J, Tang H, Duncanson L, Hancock S, Jantz P, Marselis S, Patterson PL, Qi W, Silva C. The Global Ecosystem Dynamics Investigation: high-resolution laser ranging of the Earth’s forests and topography. Sci Remote Sens. 2020;1: 100002. 10.1016/j.srs.2020.100002.

[24] Dubayah RO, Drake JB. Lidar remote sensing for forestry. J Forest. 2000;98:44–6. 10.1093/jof/98.6.44.

[25] Dudney JC, Nesmith JCB, Cahill MC, Cribbs JE, Duriscoe DM, Das AJ, Stephenson NL, Battles JJ. Compounding effects of white pine blister rust, mountain pine beetle, and fire threaten four white pine species. Ecosphere. 2020;11: e03263. 10.1002/ecs2.3263.

[26] Duncanson L, Kellner JR, Armston J, Dubayah R, Minor DM, Hancock S, Healey SP, Patterson PL, Saarela S, Marselis S, Silva CE, Bruening J, Goetz SJ, Tang H, Hofton M, Blair B, Luthcke S, Fatoyinbo L, Abernethy K, Alonso A, Andersen H-E, Aplin P, Baker TR, Barbier N, Bastin JF, Biber P, Boeckx P, Bogaert J, Boschetti L, Boucher PB, Boyd DS, Burslem DFRP, Calvo-Rodriguez S, Chave J, Chazdon RL, Clark DB, Clark DA, Cohen WB, Coomes DA, Corona P, Cushman KC, Cutler MEJ, Dalling JW, Dalponte M, Dash J, de Miguel S, Deng S, Ellis PW, Erasmus B, Fekety PA, Fernandez-Landa A, Ferraz A, Fischer R, Fisher AG, García-Abril A, Gobakken T, Hacker JM, Heurich M, Hill RA, Hopkinson C, Huang H, Hubbell SP, Hudak AT, Huth A, Imbach B, Jeffery KJ, Katoh M, Kearsley E, Kenfack D, Kljun N, Knapp N, Král K, Krůček M, Labrière N, Lewis SL, Longo M, Lucas RM, Main R, Manzanera JA, Martínez RV, Mathieu R, Memiaghe H, Meyer V, Mendoza AM, Monerris A, Montesano P, Morsdorf F, Næsset E, Naidoo L, Nilus R, O’Brien M, Orwig DA, Papathanassiou K, Parker G, Philipson C, Phillips OL, Pisek J, Poulsen JR, Pretzsch H, Rüdiger C, Saatchi S, Sanchez-Azofeifa A, Sanchez-Lopez N, Scholes R, Silva CA, Simard M, Skidmore A, Stereńczak K, Tanase M, Torresan C, Valbuena R, Verbeeck H, Vrska T, Wessels K, White JC, White LJT, Zahabu E, Zgraggen C. Aboveground biomass density models for NASA’s Global Ecosystem Dynamics Investigation (GEDI) lidar mission. Remote Sens Environ. 2022;270: 112845. 10.1016/j.rse.2021.112845.

[27] Duncanson L, Neuenschwander A, Hancock S, Thomas N, Fatoyinbo T, Simard M, Silva CA, Armston J, Luthcke SB, Hofton M, Kellner JR, Dubayah R. Biomass estimation from simulated GEDI, ICESat-2 and NISAR across environmental gradients in Sonoma County, California. Remote Sens Environ. 2020;242: 111779. 10.1016/j.rse.2020.111779.

[28] Emick E, Babcock C, White GW, Hudak AT, Domke GM, Finley AO. An approach to estimating forest biomass while quantifying estimate uncertainty and correcting bias in machine learning maps. Remote Sens Environ. 2023;295: 113678. 10.1016/j.rse.2023.113678.

[29] Fekety PA, Falkowski MJ, Hudak AT. Temporal transferability of LiDAR-based imputation of forest inventory attributes. Can J For Res. 2015;45:422–35. 10.1139/cjfr-2014-0405.

[30] Fekety PA, Falkowski MJ, Hudak AT, Jain TB, Evans JS. Transferability of Lidar-derived Basal Area and Stem Density Models within a Northern Idaho Ecoregion. Can J Remote Sens. 2018;44:131–43. 10.1080/07038992.2018.1461557.

[31] Finley AO, Banerjee S, Carlin BP. spBayes: an R package for univariate and multivariate hierarchical point-referenced spatial models. J Stat Softw. 2007;19:1–24. 10.18637/jss.v019.i04. 21494410 10.18637/jss.v019.i04 PMC3074178

[32] Finley AO, Banerjee S, Cook BD. Bayesian hierarchical models for spatially misaligned data in R. Methods Ecol Evol. 2014;5:514–23. 10.1111/2041-210X.12189.

[33] Finley AO, Banerjee S, Gelfand AE. spBayes for large univariate and multivariate point-referenced spatio-temporal data models. J Stat Softw. 2015;63:1–28. 10.18637/jss.v063.i13. 10.18637/jss.v019.i04 PMC3074178 21494410

[34] Fites-Kaufman J, Rundel P, Stephenson NL, Weixelman DA. Montane and subalpine vegetation of the Sierra Nevada and Cascade Ranges. In: Barbour Keeler-Wolf T, Schoenherr AA, Barbour MG, editors. Terrestrial vegetation of California. Berkeley: University of California Press; 2007. p. 456–501.

[35] Franklin JF, Spies TA, Pelt RV, Carey AB, Thornburgh DA, Berg DR, Lindenmayer DB, Harmon ME, Keeton WS, Shaw DC, Bible K, Chen J. Disturbances and structural development of natural forest ecosystems with silvicultural implications, using Douglas-fir forests as an example. Forest Ecol Manag Forest Ecol Next Millennium Putting Long View Pract. 2002;155:399–423. 10.1016/S0378-1127(01)00575-8.

[36] Frazer GW, Magnussen S, Wulder MA, Niemann KO. Simulated impact of sample plot size and co-registration error on the accuracy and uncertainty of LiDAR-derived estimates of forest stand biomass. Remote Sens Environ. 2011;115:636–49. 10.1016/j.rse.2010.10.008.

[37] Gobakken T, Næsset E. Assessing effects of positioning errors and sample plot size on biophysical stand properties derived from airborne laser scanner data. Can J For Res. 2009;39:1036–52. 10.1139/X09-025.

[38] Goetz SJ, Baccini A, Laporte NT, Johns T, Walker W, Kellndorfer J, Houghton RA, Sun M. Mapping and monitoring carbon stocks with satellite observations: a comparison of methods. Carbon Balance Manage. 2009;4:2. 10.1186/1750-0680-4-2. 10.1186/1750-0680-4-2 PMC2667409 19320965

[39] Goulden ML, Bales RC. Mountain runoff vulnerability to increased evapotranspiration with vegetation expansion. Proc Natl Acad Sci USA. 2014;111:14071–5. 10.1073/pnas.1319316111. 25197084 10.1073/pnas.1319316111 PMC4191806

[40] Halpern CB, Lutz JA. Canopy closure exerts weak controls on understory dynamics: a 30-year study of overstory–understory interactions. Ecol Monogr. 2013;83:221–37. 10.1890/12-1696.1.

[41] Hayhoe K, Cayan D, Field CB, Frumhoff PC, Maurer EP, Miller NL, Moser SC, Schneider SH, Cahill KN, Cleland EE, Dale L, Drapek R, Hanemann RM, Kalkstein LS, Lenihan J, Lunch CK, Neilson RP, Sheridan SC, Verville JH. Emissions pathways, climate change, and impacts on California. Proc Natl Acad Sci USA. 2004;101:12422–7. 10.1073/pnas.0404500101. 15314227 10.1073/pnas.0404500101 PMC514653

[42] Hijmans RJ. terra: spatial data analysis; 2022.

[43] Hijmans RJ. raster: geographic data analysis and modeling; 2020.

[44] Holtmeier F-K, Broll G. Sensitivity and response of northern hemisphere altitudinal and polar treelines to environmental change at landscape and local scales. Glob Ecol Biogeogr. 2005;14:395–410. 10.1111/j.1466-822X.2005.00168.x.

[45] Holtmeier F-K, Broll G. Subalpine forest and treeline ecotone under the influence of disturbances: a review. J Environ Prot. 2018;9:815–45. 10.4236/jep.2018.97051.

[46] Hooper S, Kennedy RE. A spatial ensemble approach for broad-area mapping of land surface properties. Remote Sens Environ. 2018;210:473–89. 10.1016/j.rse.2018.03.032.

[47] Houghton RA. Aboveground forest biomass and the global carbon balance. Glob Change Biol. 2005;11:945–58. 10.1111/j.1365-2486.2005.00955.x.

[48] Huang S, Ramirez C, Kennedy K, Mallory J. A new approach to extrapolate forest attributes from field inventory with satellite and auxiliary data sets. Forest Sci. 2017;63:232–40. 10.5849/forsci.16-028.

[49] Hudak AT, Fekety PA, Kane VR, Kennedy RE, Filippelli SK, Falkowski MJ, Tinkham WT, Smith AMS, Crookston NL, Domke GM, Corrao MV, Bright BC, Churchill DJ, Gould PJ, McGaughey RJ, Kane JT, Dong J. A carbon monitoring system for mapping regional, annual aboveground biomass across the northwestern USA. Environ Res Lett. 2020. 10.1088/1748-9326/ab93f9.

[50] Hurteau MD, Brooks ML. Short- and Long-Term Effects of Fire on Carbon in US Dry Temperate Forest Systems. Bisi. 2011;61:139–46. 10.1525/bio.2011.61.2.9.

[51] Jakubowski MK, Guo Q, Kelly M. Tradeoffs between lidar pulse density and forest measurement accuracy. Remote Sens Environ. 2013;130:245–53. 10.1016/j.rse.2012.11.024.

[52] Jenkins JC, Chojnacky DC, Heath LS, Birdsey RA. National-scale biomass estimators for united states tree species. Forest Sci. 2003;49:12–35. 10.1093/forestscience/49.1.12.

[53] Jiang P, Russell MB, Frelich L, Babcock C, Smith JE. Wildfires correlate with reductions in aboveground tree carbon stocks and sequestration capacity on forest land in the Western United States. Sci Total Environ. 2023;893: 164832. 10.1016/j.scitotenv.2023.164832. 37321501 10.1016/j.scitotenv.2023.164832

[54] Kane VR, Cansler CA, Povak NA, Kane JT, McGaughey RJ, Lutz JA, Churchill DJ, North MP. Mixed severity fire effects within the Rim fire: relative importance of local climate, fire weather, topography, and forest structure. For Ecol Manage. 2015;358:62–79. 10.1016/j.foreco.2015.09.001.

[55] Kane VR, McGaughey RJ, Bakker JD, Gersonde RF, Lutz JA, Franklin JF. Comparisons between field- and LiDAR-based measures of stand structural complexity. Can J For Res. 2010;40:761–73. 10.1139/X10-024.

[56] Kane VR, North MP, Lutz JA, Churchill DJ, Roberts SL, Smith DF, McGaughey RJ, Kane JT, Brooks ML. Assessing fire effects on forest spatial structure using a fusion of landsat and airborne LiDAR data in Yosemite national park. Remote Sens Environ. 2014;151:89–101. 10.1016/j.rse.2013.07.041.

[57] Kellogg K, Hoffman P, Standley S, Shaffer S, Rosen P, Edelstein W, Dunn C, Baker C, Barela P, Shen Y, Guerrero AM, Xaypraseuth P, Sagi VR, Sreekantha CV, Harinath N, Kumar R, Bhan R, Sarma CVHS. NASA-ISRO synthetic aperture radar (NISAR) mission. In: 2020 IEEE aerospace conference. Presented at the 2020 IEEE aerospace conference. p. 1–21. 10.1109/AERO47225.2020.9172638

[58] Kennedy RE, Ohmann J, Gregory M, Roberts H, Yang Z, Bell DM, Kane V, Hughes MJ, Cohen WB, Powell S, Neeti N, Larrue T, Kane J, Miller DL, Perkins J, Braaten J, Seidl R. An empirical, integrated forest biomass monitoring system. Environ Res Lett. 2018. 10.1088/1748-9326/aa9d9e.

[59] Kennedy RE, Yang ZG, Cohen WB. Detecting trends in forest disturbance and recovery using yearly Landsat time series: 1. LandTrendr—temporal segmentation algorithms. Remote Sens Environ. 2010;114:2897–910. 10.1016/j.rse.2010.07.008.

[60] Kueppers LM, Southon J, Baer P, Harte J. Dead wood biomass and turnover time, measured by radiocarbon, along a subalpine elevation gradient. Oecologia. 2004;141:641–51. 10.1007/s00442-004-1689-x. 15338416 10.1007/s00442-004-1689-x

[61] Körner C. A re-assessment of high elevation treeline positions and their explanation. Oecologia. 1998;115:445–59. 10.1007/s004420050540. 28308263 10.1007/s004420050540

[62] Lefsky MA, Cohen WB, Parker GG, Harding DJ. Lidar remote sensing for ecosystem studies. Bioscience. 2002;52:19. 10.1641/0006-3568(2002)052[0019:LRSFES]2.0.CO;2.

[63] Lefsky MA, Hudak AT, Cohen WB, Acker SA. Geographic variability in lidar predictions of forest stand structure in the Pacific Northwest. Remote Sens Environ. 2005;95:532–48. 10.1016/j.rse.2005.01.010.

[64] Liu J, Zou H-X, Bachelot B, Dong T, Zhu Z, Liao Y, Plenković-Moraj A, Wu Y. Predicting the responses of subalpine forest landscape dynamics to climate change on the eastern Tibetan Plateau. Glob Change Biol. 2021;27:4352–66. 10.1111/gcb.15727. 10.1111/gcb.15727 PMC13420788 34060175

[65] Lu DS, Chen Q, Wang GX, Liu LJ, Li GY, Moran E. A survey of remote sensing-based aboveground biomass estimation methods in forest ecosystems. Int J Digital Earth. 2016;9:63–105. 10.1080/17538947.2014.990526.

[66] Lundquist JD, Dickerson-Lange SE, Lutz JA, Cristea NC. Lower forest density enhances snow retention in regions with warmer winters: A global framework developed from plot-scale observations and modeling. Water Resour Res. 2013;49:6356–70. 10.1002/wrcr.20504.

[67] Lutz JA, Larson AJ, Swanson ME, Freund JA. Ecological importance of large-diameter trees in a temperate mixed-conifer forest. PLoS ONE. 2012;7: e36131. 10.1371/journal.pone.0036131. 22567132 10.1371/journal.pone.0036131 PMC3342248

[68] Lutz JA, Matchett JR, Tarnay LW, Smith DF, Becker KML, Furniss TJ, Brooks ML. Fire and the distribution and uncertainty of carbon sequestered as aboveground tree biomass in Yosemite and Sequoia & Kings Canyon National Parks. Land. 2017;6:10. 10.3390/land6010010.

[69] Macias-Fauria M, Johnson EA. Warming-induced upslope advance of subalpine forest is severely limited by geomorphic processes. Proc Natl Acad Sci. 2013;110:8117–22. 10.1073/pnas.1221278110. 23569221 10.1073/pnas.1221278110 PMC3657765

[70] Mallek C, Safford H, Viers J, Miller J. Modern departures in fire severity and area vary by forest type, Sierra Nevada and southern Cascades, California, USA. Ecosphere. 2013;4:1–28. 10.1890/ES13-00217.1.

[71] Mauro F, Hudak AT, Fekety PA, Frank B, Temesgen H, Bell DM, Gregory MJ, McCarley TR. Regional modeling of forest fuels and structural attributes using airborne laser scanning data in Oregon. Remote Sensing 2021;13:261. 10.3390/rs13020261.

[72] McGaughey RJ. FUSION/LDV: software for LIDAR data analysis and visualization; 2018.

[73] McKinney ST, Rodhouse T, Chow L, Chung-MacCoubrey A, Dicus G, Garret L, Irvine K, Mohren S, Odion D, Sarr D, Starcevich LA. Monitoring White Pine (*Pinus**albicaulis*, *P.**balfouriana*, *P.**flexilis*) community dynamics in the Pacific West Region—Klamath, Sierra Nevada, and Upper Columbia Basin Networks: Standard operating procedures version 1.0 (Appendix A to Narrative Version 1.0) (Natural Resource Report. NPS/PWR/NRR—2012/533). National Park Service, Fort Collins, CO; 2012.

[74] Meyer MD, Bulaon B, MacKenzie M, Safford HD. Mortality, structure, and regeneration in whitebark pine stands impacted by mountain pine beetle in the southern Sierra Nevada. Can J For Res. 2016;46:572–81. 10.1139/cjfr-2015-0464.

[75] Meyer MD, Gross S, Slaton M. Whitebark Pine inventory and monitoring protocol—region 5. USDA Forest Service Pacific Southwest Region; 2017.

[76] Meyer MD, North MP. Natural range of variation of red fir and subalpine forests in the Sierra Nevada Bioregion. Gen Tech. Rep. PSW-GTR-263. USDA Forest Service, Pacific Southwest Research Station, Albany, CA; 2019.

[77] Millar CI, Rundel P. Subalpine forests. In: Zavaleta E, Mooney HA, editors. Ecosystems of California. Berkeley: University of California; 2016.

[78] Millar CI, Westfall RD, Evenden A, Holmquist JG, Schmidt-Gengenbach J, Franklin RS, Nachlinger J, Delany DL. Potential climatic refugia in semi-arid, temperate mountains: Plant and arthropod assemblages associated with rock glaciers, talus slopes, and their forefield wetlands, Sierra Nevada, California, USA. Quatern Int. 2015;387:106–21. 10.1016/j.quaint.2013.11.003.

[79] Miller JD, Safford HD, Crimmins M, Thode AE. Quantitative evidence for increasing forest fire severity in the Sierra Nevada and southern Cascade Mountains, California and Nevada, USA. Ecosystems. 2009;12:16–32. 10.1007/s10021-008-9201-9.

[80] Morrison KD, Kolden CA. Development of a historical multi-year land cover classification incorporating wildfire effects. Land. 2014;3:1214–31. 10.3390/land3041214.

[81] Nesmith JCB, Wright M, Jules ES, McKinney ST. Whitebark and foxtail pine in yosemite, Sequoia, and Kings Canyon National Parks: initial assessment of stand structure and condition. Forests. 2019;10:35. 10.3390/f10010035.

[82] Nilsson M, Nordkvist K, Jonzén J, Lindgren N, Axensten P, Wallerman J, Egberth M, Larsson S, Nilsson L, Eriksson J, Olsson H. A nationwide forest attribute map of Sweden predicted using airborne laser scanning data and field data from the National Forest Inventory. Remote Sens Environ. 2017;194:447–54. 10.1016/j.rse.2016.10.022.

[83] Næsset E, Gobakken T, Bollandsås OM, Gregoire TG, Nelson R, Ståhl G. Comparison of precision of biomass estimates in regional field sample surveys and airborne LiDAR-assisted surveys in Hedmark County, Norway. Remote Sens Environ. 2013;130:108–20. 10.1016/j.rse.2012.11.010.

[84] Ohmann JL, Gregory MJ. Predictive mapping of forest composition and structure with direct gradient analysis and nearest-neighbor imputation in coastal Oregon, U.S.A. Can J For Res. 2002;32:725–41. 10.1139/x02-011.

[85] Painter TH, Berisford DF, Boardman JW, Bormann KJ, Deems JS, Gehrke F, Hedrick A, Joyce M, Laidlaw R, Marks D, Mattmann C, McGurk B, Ramirez P, Richardson M, Skiles SM, Seidel FC, Winstral A. The Airborne Snow Observatory: fusion of scanning lidar, imaging spectrometer, and physically-based modeling for mapping snow water equivalent and snow albedo. Remote Sens Environ. 2016;184:139–52. 10.1016/j.rse.2016.06.018.

[86] Parker GG, Harmon ME, Lefsky MA, Chen J, Pelt RV, Weis SB, Thomas SC, Winner WE, Shaw DC, Frankling JF. Three-dimensional structure of an old-growth Pseudotsuga-Tsuga canopy and its implications for radiation balance, microclimate, and gas exchange. Ecosystems. 2004;7:440–53. 10.1007/s10021-004-0136-5.

[87] Pearson JA, Fahey TJ, Knight DH. Biomass and leaf area in contrasting lodgepole pine forests. Can J For Res. 1984;14:259–65. 10.1139/x84-050.

[88] Potter DA. Forested Communities of the Upper Montane in the Central and Southern Sierra Nevada. Albany, CA: USDA Forest Service, Pacific Southwest Research Station; 1998.

[89] Powell SL, Cohen WB, Healey SP, Kennedy RE, Moisen GG, Pierce KB, Ohmann JL. Quantification of live aboveground forest biomass dynamics with Landsat time-series and field inventory data: a comparison of empirical modeling approaches. Remote Sens Environ. 2010;114:1053–68. 10.1016/j.rse.2009.12.018.

[90] Prichard SJ, Peterson DL, Hammer RD. Carbon distribution in subalpine forests and meadows of the Olympic Mountains, Washington. Soil Sci Soc Am J. 2000;64:1834–45. 10.2136/sssaj2000.6451834x.

[91] R Core Team. R: A language and environment for statistical computing. R Core Team; 2017.

[92] Roussel J-R, Auty D, Coops NC, Tompalski P, Goodbody TRH, Meador AS, Bourdon J-F, de Boissieu F, Achim A. lidR: An R package for analysis of Airborne Laser Scanning (ALS) data. Remote Sens Environ. 2020;251: 112061. 10.1016/j.rse.2020.112061.

[93] Roussel JR, Auty D. Airborne LiDAR data manipulation and visualization for forestry applications; 2023.

[94] Safford HD, Butz RJ, Bohlman GN, Coppoletta M, Estes BL, Gross SE, Merriam KE, Meyer MD, Molinari NA, Wuenschel A. Fire ecology of the North American Mediterranean-climate zone. In: Collins BM, Greenberg CH, editors. Fire ecology and management: past, present, and future of US forested ecosystems. New York: Springer; 2021. p. 337–92.

[95] Safford HD, North M, Meyer MD, North M. Climate change and the relevance of historical forest conditions, Managing Sierra Nevada forests. General Technical Report PSW-GTR-237. USDA Forest Service. Pacific Southwest Research Station, Albany, CA; 2012.

[96] Schwartz MW, Butt N, Dolanc CR, Holguin A, Moritz MA, North MP, Safford HD, Stephenson NL, Thorne JH, van Mantgem PJ. Increasing elevation of fire in the Sierra Nevada and implications for forest change. Ecosphere. 2015;6:art121. 10.1890/ES15-00003.1.

[97] Sherrill KR, Lefsky MA, Bradford JB, Ryan MG. Forest structure estimation and pattern exploration from discrete-return lidar in subalpine forests of the central Rockies. Can J For Res. 2008;38:2081–96. 10.1139/X08-059.

[98] Slaton MR, MacKenzie M, Kohler T, Ramirez CM. Whitebark Pine recruitment in Sierra Nevada driven by range position and disturbance history. Forests. 2019;10:455. 10.3390/f10050455.

[99] Smith WB. Forest inventory and analysis: a national inventory and monitoring program. Environ Pollut. 2002;116:S233–42. 10.1016/S0269-7491(01)00255-X. 11833910 10.1016/s0269-7491(01)00255-x

[100] Smithers BV, North MP, Millar CI, Latimer AM. Leap frog in slow motion: divergent responses of tree species and life stages to climatic warming in Great Basin subalpine forests. Glob Change Biol. 2018;24:e442–57. 10.1111/gcb.13881. 10.1111/gcb.13881 28850759

[101] Stevens DL, Olsen AR. Spatially balanced sampling of natural resources. J Am Stat Assoc. 2004;99:262–78. 10.1198/016214504000000250.

[102] Stovall AEL, Fatoyinbo T, Thomas NM, Armston J, Ebanega MO, Simard M, Trettin C, Obiang Zogo RV, Aken IA, Debina M, Me Kemoe AM, Assoumou EO, Kim JS, Lagomasino D, Lee S-K, Ndong Obame JC, Voubou GD, Essono CZ. Comprehensive comparison of airborne and spaceborne SAR and LiDAR estimates of forest structure in the tallest mangrove forest on earth. Sci Remote Sens. 2021;4: 100034. 10.1016/j.srs.2021.100034.

[103] Stovall AEL, Shugart HH, Yang X. Reply to “Height-related changes in forest composition explain increasing tree mortality with height during an extreme drought.” Nat Commun. 2020;11:3401. 10.1038/s41467-020-17214-4. 32636374 10.1038/s41467-020-17214-4 PMC7340790

[104] Tenneson K, Patterson MS, Mellin T, Nigrelli M, Joria P, Mitchell B. Development of a regional lidar-derived above-ground biomass model with Bayesian model averaging for use in Ponderosa pine and mixed conifer forests in Arizona and New Mexico, USA. Remote Sens. 2018;10:1–28. 10.3390/rs10030442.

[105] Tojal L-T, Bastarrika A, Barrett B, Sanchez Espeso JM, Lopez-Guede JM, Graña M. Prediction of aboveground biomass from low-density LiDAR data: validation over *P.**radiata* data from a Region North of Spain. Forests. 2019;10L:819. 10.3390/f10090819.

[106] Tranquillini W. Physiological ecology of the alpine timberline: tree existence at high altitudes with special reference to the European alps. Berlin: Springer-Verlag; 1979.

[107] US Fish and Wildlife Service. Endangered and Threatened Wildlife and Plants; Threatened Species Status With Section 4 (d) Rule for Whitebark Pine (*Pinus**albicaulis*) (Federal Register No. 87 FR 76882); 2022.

[108] USDA Forest Service. Field instructions for the annual inventory of California, Oregon, and Washington. USDA Pacific Northwest Research Station; 2022.

[109] USDA Forest Service. Existing vegetation—CALVEG [ESRI personal geodatabase]. McClellan: USDA Forest Service Pacific Southwest Region; 2018.

[110] USDA Forest Service, n.d. CALVEG Vegetation Descriptions, CALVEG zones 3, 4, and 9. USDA Forest Service Pacific Southwest Region [WWW Document]. https://www.fs.usda.gov/detail/r5/landmanagement/resourcemanagement/?cid=stelprdb5347192

[111] Urbazaev M, Thiel C, Cremer F, Dubayah R, Migliavacca M, Reichstein M, Schmullius C. Estimation of forest aboveground biomass and uncertainties by integration of field measurements, airborne LiDAR, and SAR and optical satellite data in Mexico. Carbon Balance Manag. 2018. 10.1186/s13021-018-0093-5. 10.1186/s13021-018-0093-5 PMC5821638 29468474

[112] Wilson BT, Woodall CW, Griffith DM. Imputing forest carbon stock estimates from inventory plots to a nationally continuous coverage. Carbon Balance Manage. 2013;8:1. 10.1186/1750-0680-8-1. 10.1186/1750-0680-8-1 PMC3564769 23305341

[113] Woodall CW, Heath LS, Domke GM, Nichols MC. Methods and equations for estimating aboveground volume, biomass, and carbon for trees in the U.S. forest inventory, 2010 (No. NRS-GTR-88). U.S. Department of Agriculture, Forest Service, Northern Research Station, Newtown Square, PA; 2011. 10.2737/NRS-GTR-88

[114] Young DJN, Slaton MR, Koltunov A. Temperature is positively associated with tree mortality in California subalpine forests containing whitebark pine. Ecosphere. 2023;14: e4400. 10.1002/ecs2.4400.

[115] Zhao F, Guo Q, Kelly M. Allometric equation choice impacts lidar-based forest biomass estimates: a case study from the Sierra National Forest, CA. Agric For Meteorol. 2012;165:64–72. 10.1016/j.agrformet.2012.05.019.

[116] Zolkos SG, Goetz SJ, Dubayah R. A meta-analysis of terrestrial aboveground biomass estimation using lidar remote sensing. Remote Sens Environ. 2013;128:289–98. 10.1016/j.rse.2012.10.017.

