# Supplementary material for: Improved aboveground biomass estimation and regional assessment with aerial lidar in California’s subalpine forests
Source: Carbon Balance Manag. 2024 Dec 20;19:41. doi: 10.1186/s13021-024-00286-w (PMC11662458; doi:10.1186/s13021-024-00286-w)
Supplement: Supplementary file 1 — Supplementary Material 1. [file 13021_2024_286_MOESM1_ESM.docx]

**Supplement**

Winsemius, S., Babcock, C., Kane, V.R., Bormann, K.J., Safford, H.D., Jin, Y. Improved aboveground biomass estimation and regional assessment with aerial lidar in California's subalpine forests. Carbon Balance and Management.

Corresponding author: swinsemius@ucdavis.edu

**Contents**

Supplement A:

Random forest analysis 2

Supplement B:

Table S1: Lidar specifications 6

Figure S1: Histogram of geostatistical model values 7

**Supplement A: Random forest analysis**

**Methods**

Random forest models are a commonly used method for nonparametric prediction, designed to avoid overfitting data (Breiman, 2001). We applied a random forest model to the full list of 17 lidar predictors and calculated AGB in a 10-fold cross-validation. Random forests use an ensemble of decision trees and are commonly used for non-linear prediction because they balance bias and variance (Breiman, 2001; Hultquist et al., 2014; Segal, 2004). The “bagging” ensemble learning method, in which bootstrapped samples construct decision trees (the number of which is determined by the ntree parameter) that do not depend on each other, is combined with an independently determined sample of variables at each node (the mtry parameter specifies how many variables are considered), and a majority vote determines the final prediction (Liaw and Wiener, 2002). Random predictor selection limits bias in the model, while the ensemble use of many trees (the “forest”) lowers variance (Prasad et al., 2006).

We used the randomForest package in R (Liaw and Wiener, 2002) and tested multiple combinations of mtry and ntree variables. Little variation was found with different parameters, with slightly better model performance with five variables per node and 100 trees. In order to calculate standard deviation and confidence intervals in a comparable way to the Bayesian models, we constructed a 10-fold cross-validation with the model trained on 90% of the field data and predicted 100 output trees for the 10% testing data. The process was repeated for each fold and standard deviation was calculated across the 100 trees; although nonparametric models do not guarantee Gaussian distributions, 95% confidence intervals were approximated as standard deviation multiplied by 1.96.

Table S.A1. Uncertainty measurements from the 10-fold cross-validations for the null spatial, geostatistical, and random forest models. We report five measures of model accuracy and uncertainty: root mean square error (RMSE), relative squared error (RSE), R squared (R^2^), 95% coverage probability (the percent of measured plot values that fall within the calculated 95% credible interval (null and spatial) or confidence interval (random forest), and the mean width of the 95% credible interval/confidence interval.

|  | Null spatial | Geostatistical | Random forest |
| --- | --- | --- | --- |
| *RMSE* (Mg/ha) | 96.38 | 49.06 | 49.03 |
| *RSE* | 105.76% | 27.41% | 27.37% |
| *R^2^* | 0.01 | 0.73 | 0.73 |
| 95% coverage probability | 93.61% | 94.72% | 92.4% |
| Mean 95% CI width (Mg/ha) | 326.0 | 152.7 | 164.3 |


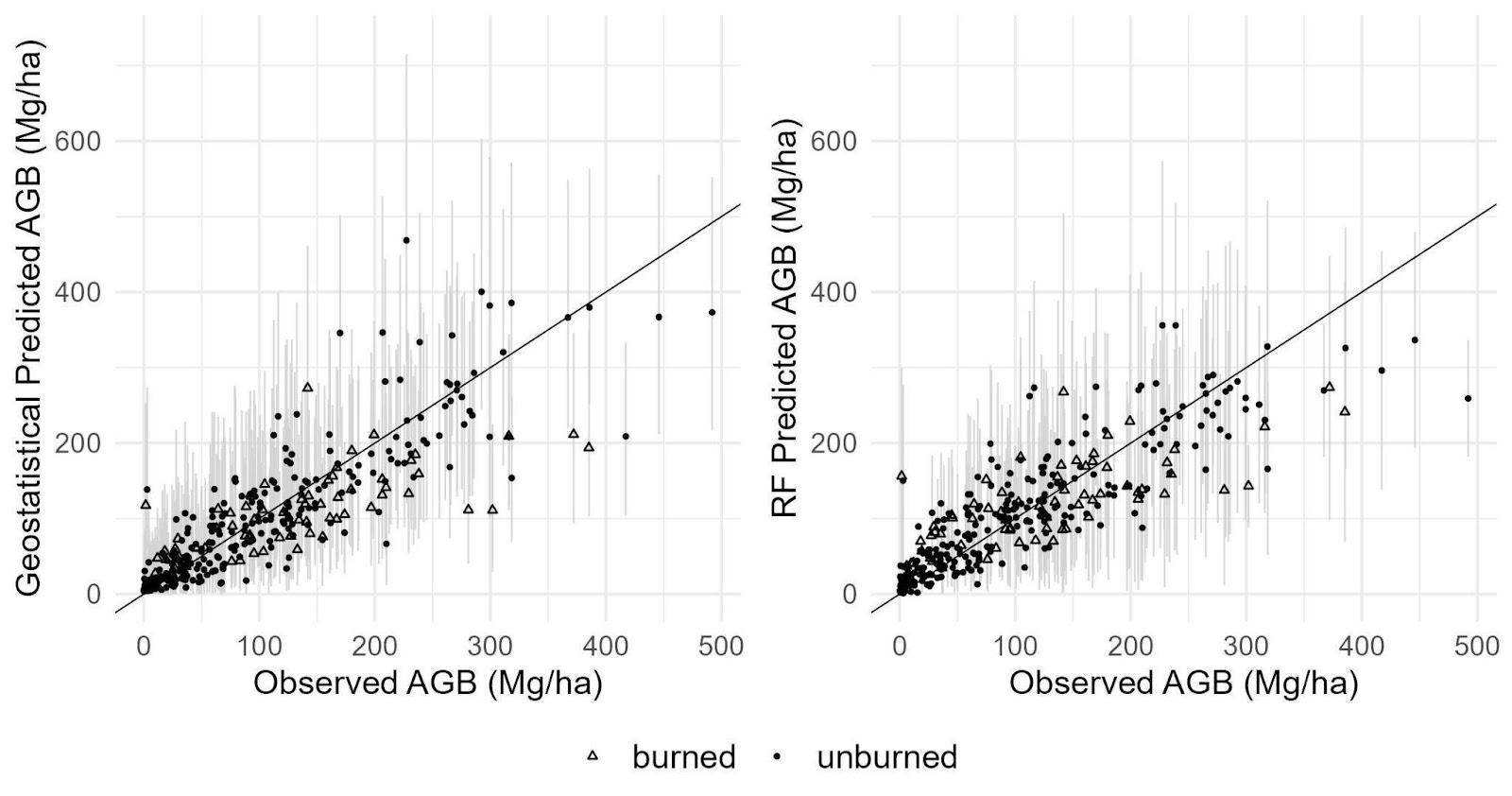


Figure S.A1. Cross-validation results comparing field-measured AGB with the model predicted AGB for the geostatistical and random forest (RF) models. Vertical gray lines show the 95% credible interval (geostatistical) or confidence interval (RF), which is the range containing the central 95% of predicted values for each plot.

**Results and Discussion**

The random forest model had similar RMSE, RSE, and R^2^ values after the 10-fold cross-validation as the Bayesian geostatistical model (Table S.A1). The 95% coverage probability was also similar to the geostatistical model, at 92.4% (Table S.A1, Fig. S.A1). The most important variables were similar to the variables chosen through our variable selection procedure: canopy cover, canopy rumple, and mean height ranking as first, second, and fourth in permutation importance, and canopy rumple and mean height ranked as the most important for node impurity.

When the prediction was applied to a test region and standard deviation calculated from the estimate from each tree, a map of standard deviation from the random forest model shows much more noise compared with the geostatistical model (Figure S.A2). This could indicate lower interpretability of variance measures, especially given the Gaussian assumption in our method for determining standard deviation and confidence intervals, which do not apply to random forests.

The random forest model appears to underpredict high observed AGB, which could indicate more saturation in the random forest model than the geostatistical model (Fig. S.A1). This could mean the geostatistical method is more robust at the limits of the model where there are fewer points, despite the fewer lidar metrics included in the model.

Another drawback of random forest models is the inability to aggregate prediction across areas to determine overall estimates and uncertainty, as we did for management units with the geostatistical model. The flexibility of the geostatistical model to jointly predict over any given region allows for greater management and policy applications of outputs.

Geostatistical Random forest

b


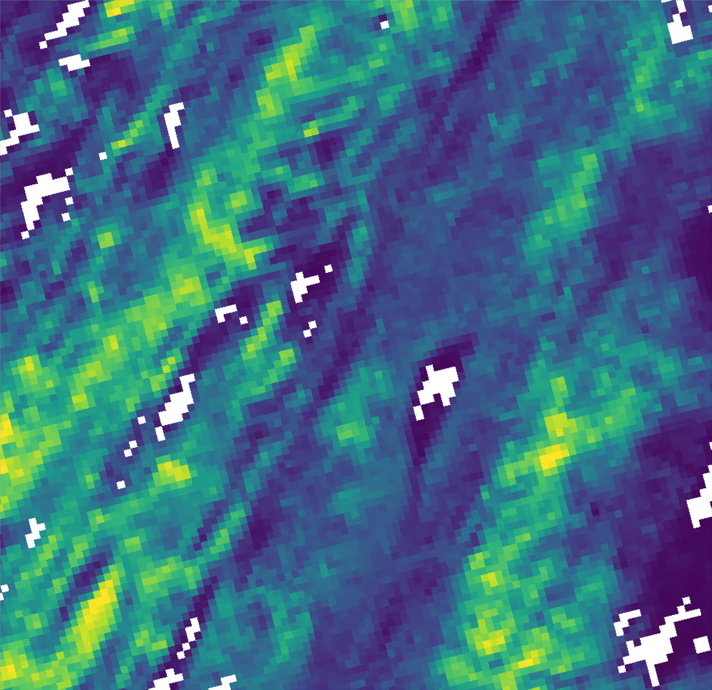

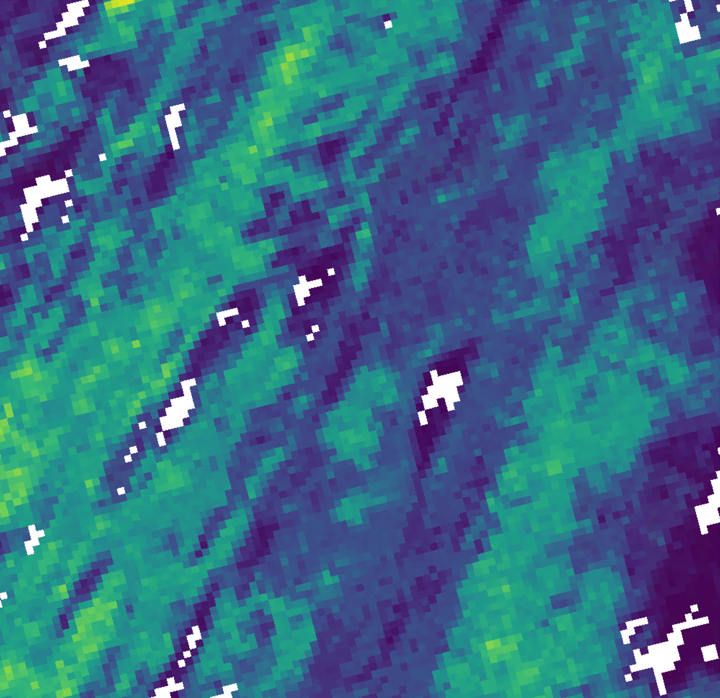


a


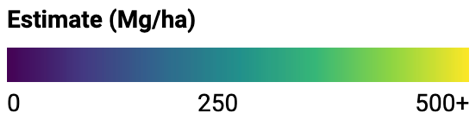


d

c


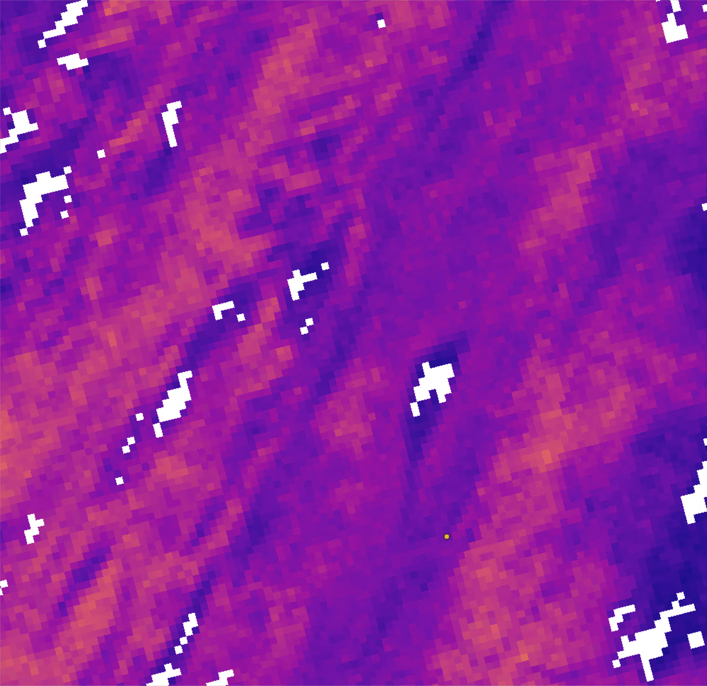

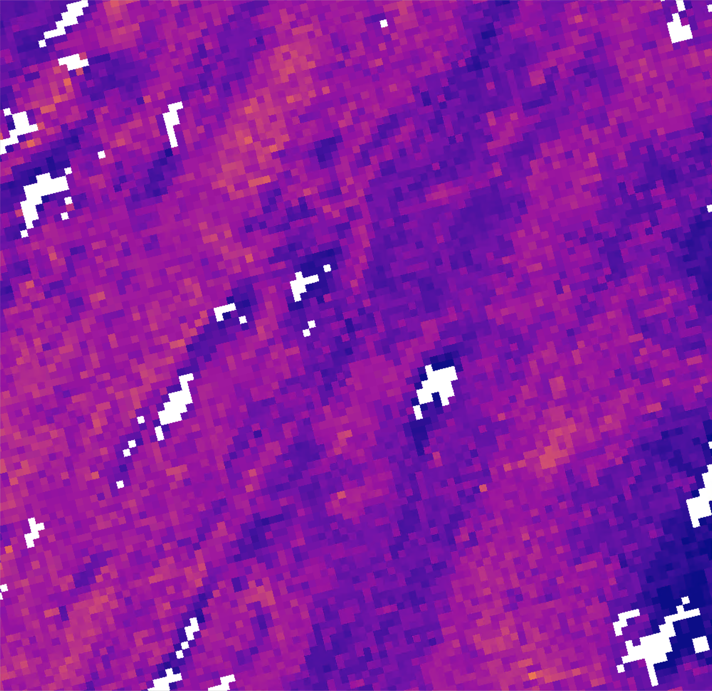


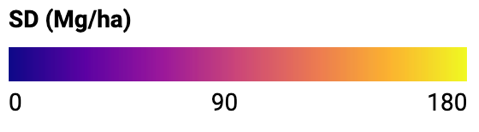


Figure S.A2. Maps showing a subset region with the estimate (a and b) and standard deviation (c and d) for the geostatistical model (a and c) and the random forest model (b and d).

**References**

Breiman L. Random Forests. European Journal of Mathematics. 2001;45:5–32.

Hultquist C, Chen G, Zhao K. A comparison of Gaussian process regression, random forests and support vector regression for burn severity assessment in diseased forests. Remote Sens Lett. 2014;5:723–32. <https://doi.org/10.1080/2150704X.2014.963733>.

Liaw A, Wiener M. Classification and regression by randomForest. R News. 2002;2:18–22.

Prasad AM, Iverson LR, Liaw A. Newer classification and regression tree techniques: bagging and random forests for ecological prediction. Ecosystems. 2006;9:181–99. <https://doi.org/10.1007/s10021-005-0054-1>.

Segal MR. Machine learning benchmarks and random forest regression (eScholarship Repository). University of California; 2004.

**Supplement B.**

Table S1. Summary of lidar specifications for each acquisition. Not all values were available from the data collector.

| **Data collector** | **Acquisition region** | **Year** | **Sensor** | **Average pulse density (pls/m2)** | **Swath Angle** | **Swath width overlap (%)** |
| --- | --- | --- | --- | --- | --- | --- |
| ASO | Carson | 2021 | Riegl VQ1560ii-s | 8 | 58 | 50 |
| ASO | Kaweah | 2018 | Riegl VQ1560 | 3.5 | 58 | 20 |
| Watershed Sciences | Kern Plateau | 2011 | ALS50 ii | 6 | 14 | 50 |
| ASO | Kings | 2014 | Riegl VQ1560 | 2 | 58 | 20 |
| ASO | Kings | 2015 | Riegl VQ1560 | 8 | 58 | 20 |
| Quantum Spatial, Inc. Corvallis for USGS | Merced | 2019 | Riegl VQ1560i | 6.5 | 58.5 | 55 |
| ASO | Mono | 2014 | Riegl VQ1560 | 2.8 | 58 | 20 |
| Contractor for ASO | Mono | 2020 | Riegl VQ1560ii-s | 9.5 | 58 | unavailable |
| ASO | San Joaquin | 2016 | Riegl VQ1560 | 8 | 58 | 20 |
| ASO | Truckee | 2018 | Riegl VQ1560 | 27 | 58 | 50 |
| ASO | Truckee | 2021 | Riegl VQ1560ii-s | 9 | 58 | 50 |
| Quantum Spatial, Inc. Corvallis for USGS | Tuolumne | 2019 | Riegl VQ1560i | 6.1 | 58.5 | 55 |
| ASO | Tuolumne | 2019 | Riegl VQ1560 | 2.3 | 58 | 20 |
| ASO | Yuba | 2018 | Riegl VQ1560i | 4.34 - 8.0 | 29 | 55 |


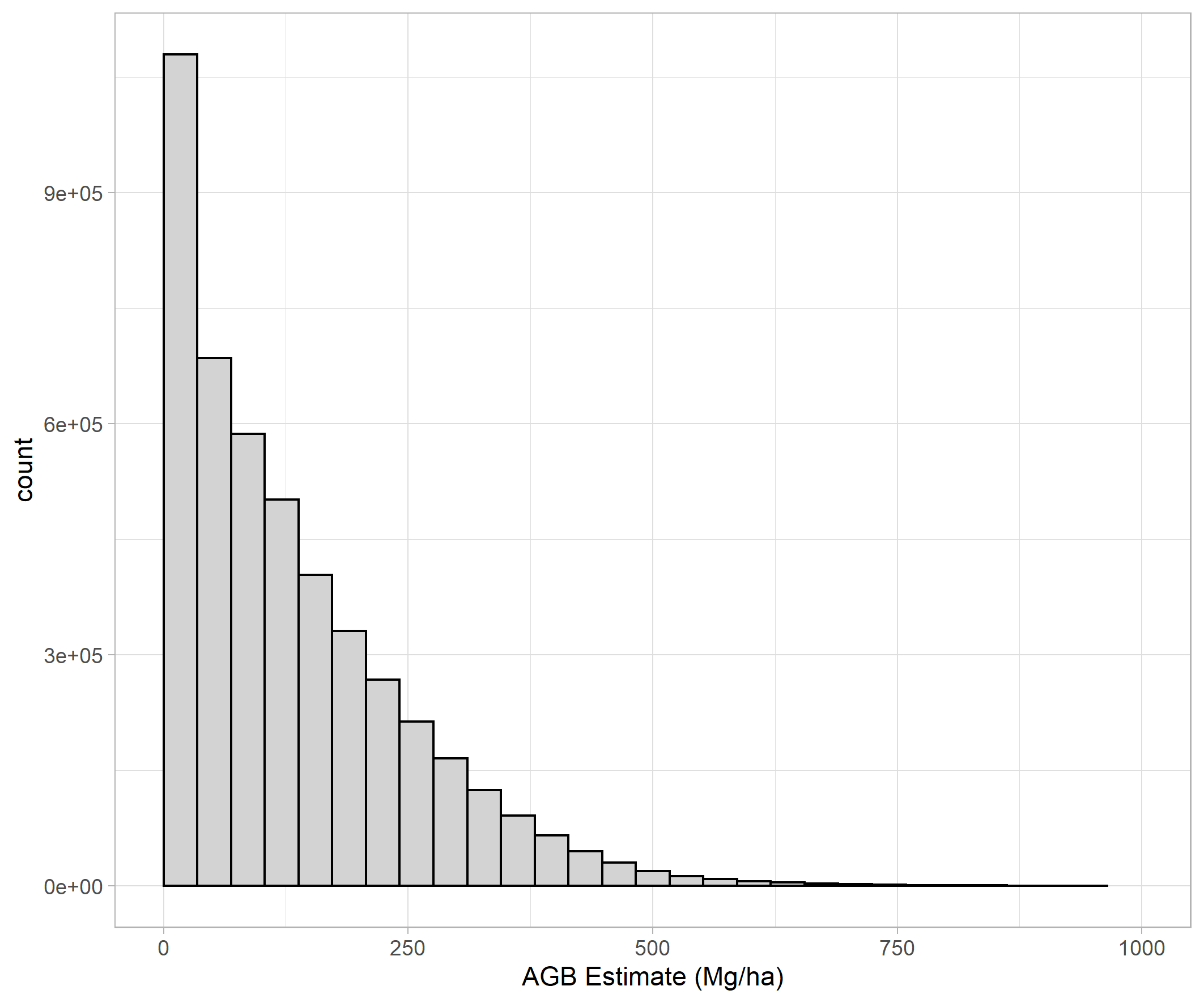


Figure S1. Histogram of values from the geostatistical model. Quantiles of the estimate are 25%: 38.2, 50%: 101.9, 75%: 196.0, 95%: 367.0, and 99% 509.5. At the high end of the range, 0.1% of pixels fell above 745.5 Mg/ha, with a maximum of 1330.9 Mg/ha.
